# Support‐Induced Interfacial Effects Steer Methanol Selectivity in CO_2_ Electroreduction by Immobilized Cobalt Phthalocyanine

**DOI:** 10.1002/anie.202521683

**Published:** 2025-11-22

**Authors:** Ke Ye, Min Hu, Guozhen Zhang, Mårten S. G. Ahlquist

**Affiliations:** ^1^ Division of Theoretical Chemistry and Biology KTH Royal Institute of Technology Stockholm 10691 Sweden; ^2^ Hefei National Research Center for Physical Sciences at the Microscale School of Chemistry and Materials Science University of Science and Technology of China Hefei 230026 China

**Keywords:** CO_2_ reduction reaction, Electrocatalysis, Immobilized cobalt phthalocyanine, Methanol

## Abstract

Achieving product selectivity in multistep electrocatalysis requires delicately tuning the stability of key reaction intermediates to modulate the energetics of competing pathways. The conversion of CO_2_ by cobalt phthalocyanine (CoPc) presents a stark puzzle in this context; while solution‐phase CoPc exclusively produces CO, its immobilization on carbon nanotube support triggers a substantial and unusual switch to methanol. Here, using multiscale simulations, we resolve this outstanding puzzle. We demonstrate that the carbon support is not a passive anchor but an active modulator of the catalytic environment. It functions by shielding one face of the CoPc molecule from the aqueous solvent, which substantially lowers the kinetic barrier for protonation while having little effect on CO desorption, thereby activating methanol production. Interfacial electric fields (EF) and cations further enhance this effect. Our findings establish support‐induced desolvation as a new, rational design guideline for kinetically steering complex electrocatalytic reactions, providing a clear mechanistic basis for the unique reactivity of heterogenized molecular catalysts.

## Graphical abstract

Immobilizing CoPc on a carbon support switches its CO_2_ reduction selectivity from CO to CH_3_OH. Multiscale simulations reveal that the support actively modulates the catalytic environment by inducing desolvation of bottom layer of CoPc, which lowers the protonation barrier while leaving CO desorption nearly unaffected.

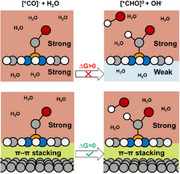

A central tenet in catalyst design is achieving proper stabilization of key reaction intermediates to rebalance the relative energetics of competing reaction pathways toward a desired product.^[^ ^1^, ^2^, ^3^, ^4^ ^]^ The selective electroreduction of CO_2_ to methanol (CH_3_OH) by cobalt phthalocyanine (CoPc) presents a stark and puzzling manifestation of this principle. In a homogeneous solution, CoPc is a competent but unselective catalyst, predominantly yielding carbon monoxide (CO).^[^ ^5^, ^6^, ^7^, ^8^ ^]^ A transformative breakthrough occurred when it was discovered that immobilizing CoPc specifically on a carbon nanotube support dramatically switches the selectivity to achieve remarkable activity for CH_3_OH production.^[^ ^9^ ^]^ This finding has since garnered intense attention,^[^ ^10^, ^11^, ^12^, ^13^, ^14^ ^]^ yet it raises a critical question that has challenged the field: Why does the graphene support, in particular, enable this profound shift in product selectivity?

While subsequent studies have identified contributing factors such as macrocycle distortion,^[^ ^15^, ^16^, ^17^, ^18^ ^]^ cation effects, and local EF,^[^ ^19^, ^20^, ^21^, ^22^, ^23^ ^]^ a fundamental mechanism explaining the unique role of the graphitic interface is badly needed because this gap in understanding hinders the development of a rational design strategy for new selective catalysts. Recent theoretical advances have provided critical insights into the elementary steps of this reaction. ^[^ ^24^, ^25^, ^26^, ^27^, ^28^, ^29^ ^]^ For instance, Hammes–Schiffer and co‐workers elegantly demonstrated that the graphitic support can act as an electron reservoir, facilitating concerted proton‐coupled electron transfer to CO_2_ reduction intermediates.^[^ ^24^ ^]^ This work brilliantly illuminates the electronic mechanism by which specific protonation‐reduction steps occur. However, it does not resolve the central thermodynamic puzzle of selectivity: why does the mere presence of the graphene support so dramatically re‐balance the kinetic competition between *CO protonation and the facile, thermodynamically favored CO desorption? An explanation for the overall shift in product preference from CO to methanol remains elusive, requiring a focus not just on the electronic pathway of a single step, but on the interfacial environment's influence on the entire energetic landscape.

In this study, we employ multiscale simulations, integrating density functional theory (DFT) and classical molecular dynamics (MD), to dissect the CO_2_RR mechanism on immobilized CoPc. By analyzing the potential‐dependent charge states of the *CO intermediate (([*CO]^0^, [*CO]^−^, and [*CO]^2−^) and meticulously mapping the free energy landscapes of competing CO desorption and *CO protonation pathways (*CHO formation), we elucidate the decisive role of the interface. Our simulations demonstrate that the interface modifies the energetics of anionic *CO species, substantially selectively lowering the kinetic barrier for their protonation relative to desorption, thereby activating the methanol pathway. This work provides a detailed atomistic mechanism for the CoPc system and illuminates an alternative strategy for rationally controlling product selectivity; manipulating the electrocatalytic interface to kinetically steer reaction outcomes by selectively destabilizing key charged intermediates.

## CO_2_ Reduction to Adsorbed CO (*CO)

**Figure 1:**
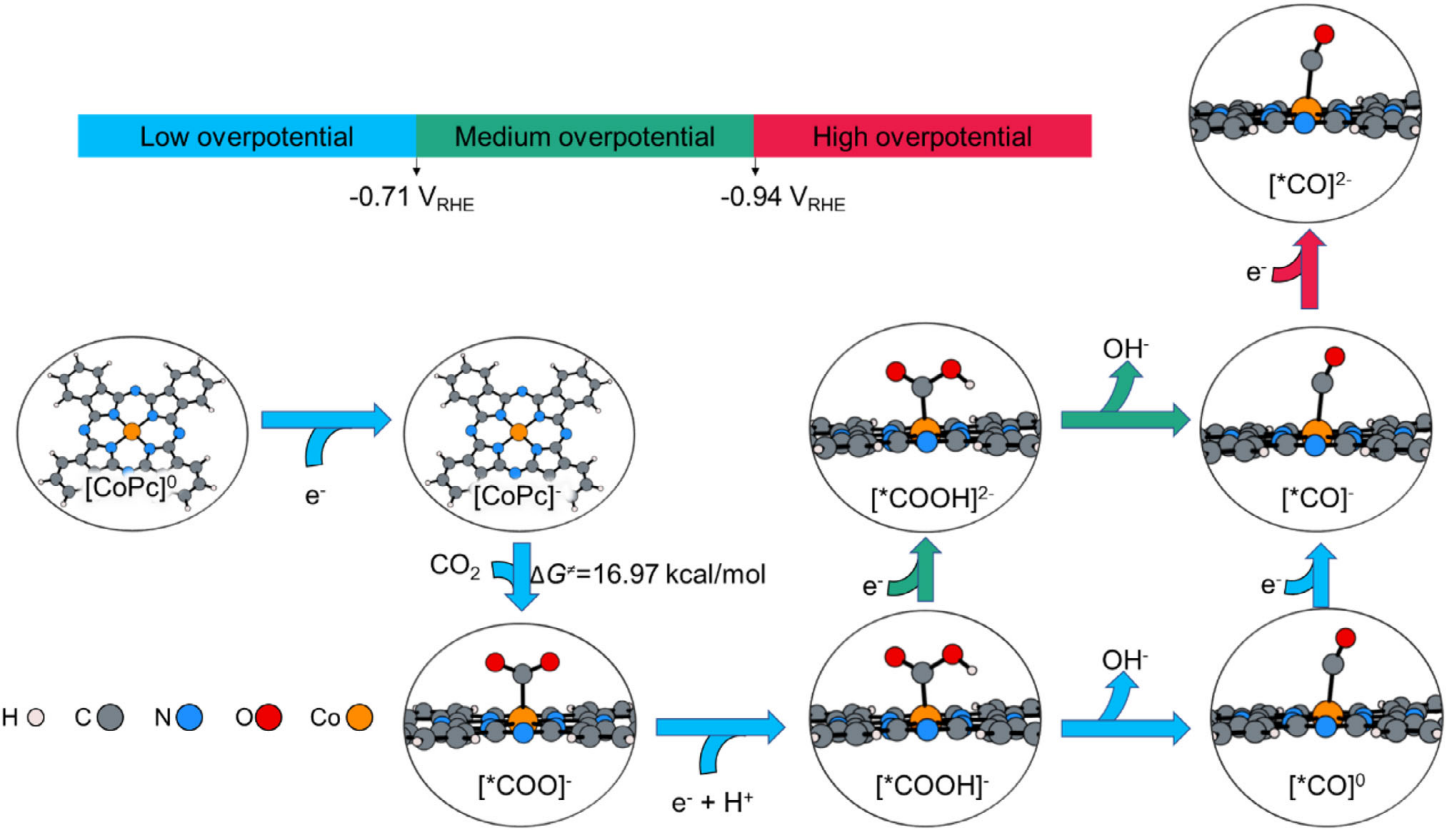
Mechanism of CO_2_ reduction to adsorbed CO catalyzed by CoPc in pure aqueous solution.

Extensive experiments have identified adsorbed CO as a key intermediate in CoPc‐catalyzed CO_2_ reduction to CH_3_OH.^[^ ^19^, ^20^, ^30^ ^]^ Our calculations show that CO_2_ reduction to *CO by CoPc in aqueous solution proceeds through several key steps (Figure 1). The neutral CoPc (Co(II)) is readily reduced to Co(I) within the experimental potential range,^[^ ^20^ ^]^ consistent with in situ X‐ray absorption results identifying Co(I) as the catalyst's resting state.^[^ ^30^ ^]^ CO_2_ then adsorbs onto the Co(I) center with a barrier of 16.97 kcal mol^−1^ to form *COO, which undergoes a proton‐electron transfer to yield [*COOH]^−^.

The formation of adsorbed CO (*CO) proceeds via different pathways depending on the applied potential and the catalyst's proximity to the electrode. At low overpotentials (U > ‐0.71 V_RHE_), especially for CoPc suspended in solution, [*COOH]^−^ likely dissociates OH^−^ to form neutral [*CO]^0^ intermediate (more detail in Figures S1–S4), which is then rapidly reduced to [*CO]^−^ (Figure 1, blue pathway). At moderate overpotentials (‐0.94 < U < ‐0.71 V_RHE_) or when CoPc is immobilized on the electrode, faster electron transfer^[^ ^24^ ^]^ may enable direct conversion of [*COOH]^−^ to [*CO]^−^ or proceed via a doubly reduced [*COOH]^2−^ species (Figure 1, green pathway). At high overpotential (U < ‐0.94 V_RHE_), further reduction leads to the formation of [*CO]^2−^ species (Figure 1, red pathway).

Overall, our calculations reveal that the rate‐determining step (RDS) for CO_2_ reduction to *CO catalyzed by CoPc in aqueous solution is the CO_2_ adsorption, consistent with experimental observations indicating that CO_2_ adsorption, rather than proton transfer, is the RDS for *CO formation.^[^ ^24^ ^]^ Within the experimentally applied potential range, *CO can exist in three charge states: [*CO]^0^, [*CO]^−^, and [*CO]^2−^,^[^ ^5^ ^]^ which are stabilized progressively at higher overpotentials. Experimentally, when CoPc is immobilized on CNTs, methanol formation is first detected at approximately –0.82 V_RHE_,^[^ ^9^ ^]^ corresponding to the emergence of the [*CO]^−^ intermediate. The selectivity toward CH_3_OH reaches its maximum at –0.95 V_RHE_, associated with the [*CO]^2−^ intermediate.^[^ ^9^, ^20^ ^]^ Throughout the entire process from CO_2_ adsorption to *CO formation, the Co center remains in the Co(I) oxidation state, consistent with experimental evidence identifying Co(I) as the catalytically active species in CoPc‐based CO_2_RR catalysts^[^ ^13^, ^31^, ^32^, ^33^ ^]^

## The Competition between CO Desorption and Protonation of *CO

**Figure 2:**
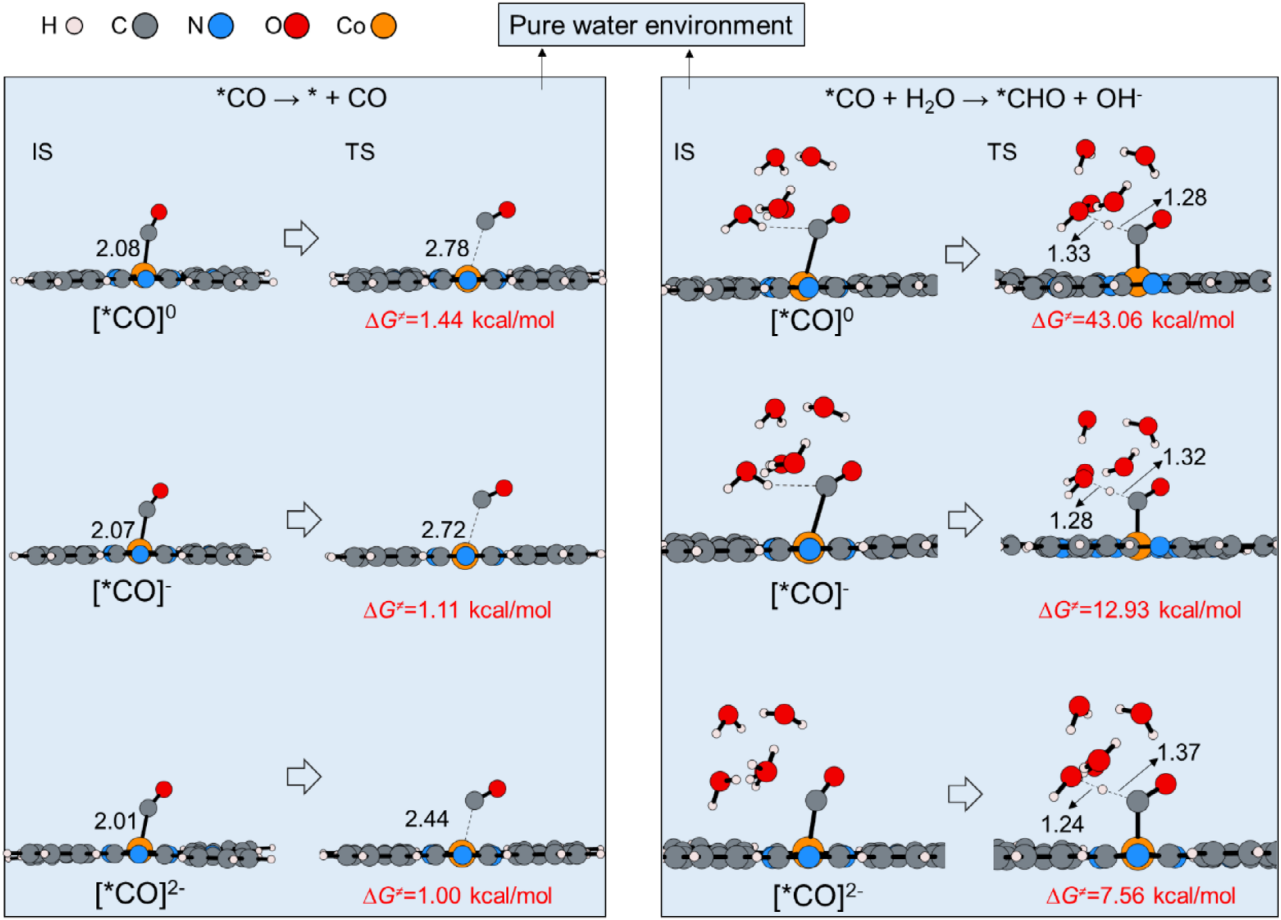
Competition between CO and CH_3_OH formation from adsorbed CO in different charge states. Black numbers: bond lengths (in Å); red numbers: Gibbs free energy barriers.

Once the *CO intermediate forms, its fate is determined by the competition between two nonelectron‐transfer steps: 1) desorption to release CO gas and 2) protonation to yield *CHO, the precursor to CH_3_OH. In a fully solvated aqueous environment, our calculations show that this competition is decisively won by desorption. For all three charge states ([*CO]^0^, [*CO]^−^, and [*CO]^2−^), the energy barrier for CO desorption is negligible (<1.5 kcal mol^−1^), indicating a spontaneous and rapid process (Figure 2), consistent with experimental evidence that *CO is a highly labile intermediate.^[^ ^10^, ^30^, ^34^ ^]^ In contrast, protonation of [*CO]^0^ requires a prohibitive barrier of 43.06 kcal mol^−1^. Although additional electron transfer to form [*CO]^−^ and [*CO]^2−^ reduces this barrier to 12.93 and 7.56 kcal mol^−1^, respectively, these values remain substantially higher than the nearly barrierless desorption. Consequently, under homogeneous aqueous conditions, CO release is overwhelmingly favored. This provides a clear atomistic basis for the experimental observation that CoPc in solution exclusively produces CO.

**Figure 3:**
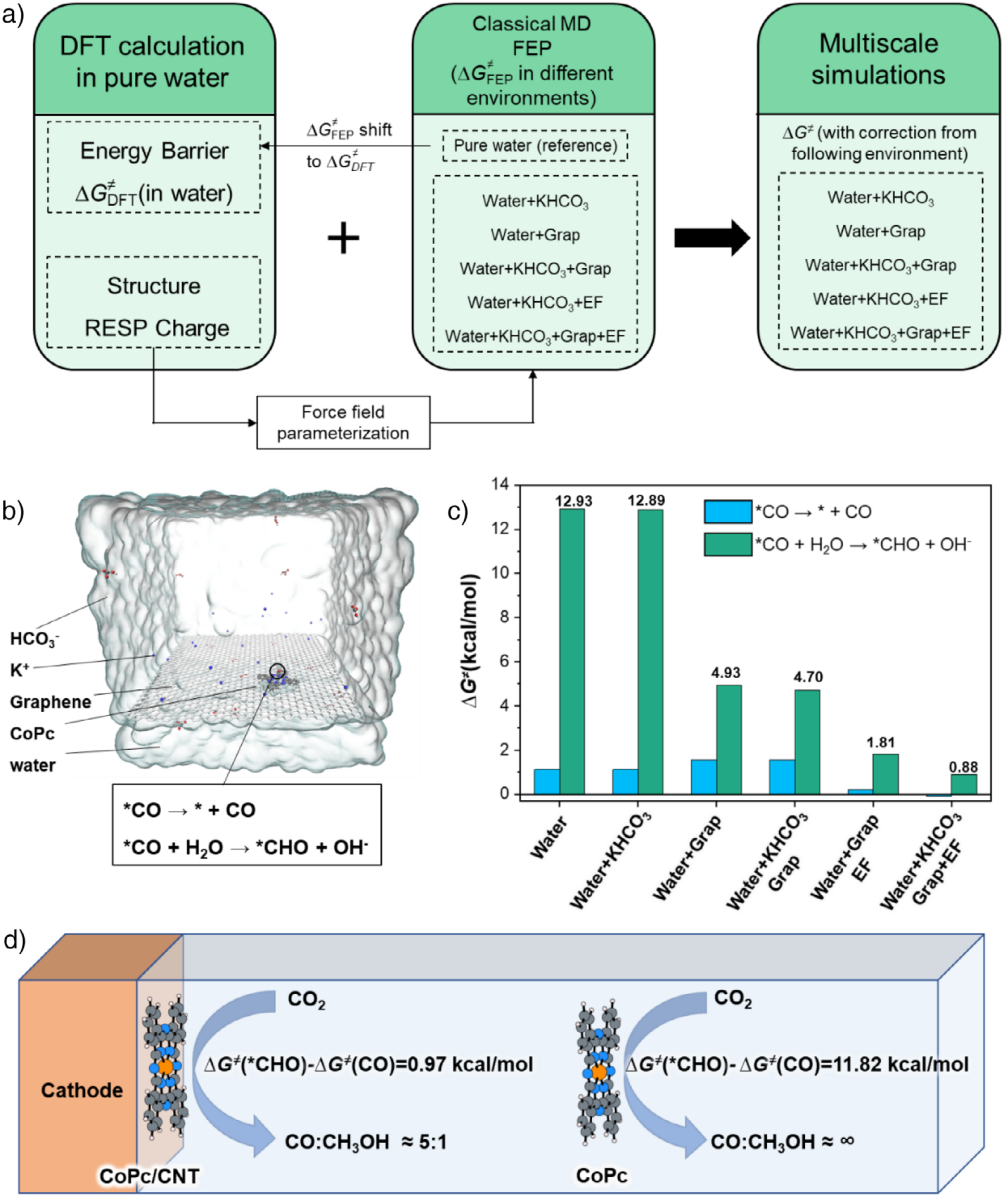
a) The workflow of multiscale simulation. b) Schematic diagram of the model used in FEP simulations. c) Influence of different EDL components on the energy barriers of CO desorption and [*CO]^−^ protonation. d) Comparison of energy barriers for CO protonation and CO desorption on CoPc in solution and on CoPc/CNT.

When CoPc is immobilized on graphene within a simulated electric double layer (EDL), the selectivity landscape changes dramatically. Our free energy perturbation (FEP) simulations show that the full interfacial environment (CoPc/graphene in KHCO_3_ solution with an applied EF) fundamentally alters the energetics of the competing pathways (Figure 3).

## The EDL Effect on the Competition Between CO Desorption and Protonation of [*CO]^−^

We use a multiscale modeling approach to investigate how the EDL influences the selectivity of CoPc‐catalyzed CO_2_ reduction by examining the energy barriers for CO desorption and protonation. As shown in Figure 3a, first, DFT calculations were performed to determine these barriers in pure water. Based on the DFT‐optimized structures and charge distributions, force fields were developed for MD simulations. The free energy profiles obtained from MD in water (∆*G*_FEP_) is aligned with the DFT results (∆*G*_DFT_) to enable consistent comparison across six environments: 1) pure water, 2) KHCO_3_ solution, 3) CoPc/G in pure water, 4) CoPc/G in KHCO_3_ solution, 5) CoPc/G under applied EF, and 6) the actual EDL environment (CoPc/G in KHCO_3_ solution with EF). To ensure sufficient sampling, a large EDL model (69.1 × 68.4 × 66.5 Å^3^) was constructed (Figure 3b, see Supporting Information for details). The FEP results are shown in Figure 3c; the CO desorption barrier remains nearly unchanged from pure water to the actual EDL environment, whereas the protonation energy barrier dramatically decreases from 12.93 to 0.88 kcal mol^−1^. As shown in Figure 3d, this EDL effect makes the two pathways kinetically competitive, predicting a CO:CH_3_OH ratio of approximately 5:1—consistent well with experimental observations.^[^ ^9^, ^20^ ^]^

By systematically dissecting the interface components, we identified the graphene support as the primary contributor to the observed change. Immobilizing CoPc on graphene alone, the protonation barrier decreases from 12.93 to 4.93 kcal mol^−1^. The addition of KHCO_3_ or an applied EF further lowers the barrier to 4.70 and 1.81 kcal mol^−1^, respectively. This indicates that the support does more than serve as an electronic anchor or conductor; it actively modulates the catalytic environment. When graphene, EF, and KHCO_3_ coexist, the electrolyte mildly facilitates proton transfer, in line with experimental findings that cations can promote CH_3_OH formation.^[^ ^20^ ^]^ We also evaluated the free‐energy barrier in the actual EDL with LiHCO_3_ as the electrolyte. In the LiHCO_3_‐based EDL, the proton‐transfer barrier is slightly lower than with K⁺, consistent with experiments showing that smaller cations enhance CH_3_OH formation.^[^ ^19^ ^]^ We further examined the EDL's effect on [*CO]^2−^ and found that it markedly enhances protonation while slightly inhibiting CO desorption (Figure S5). This partially explains the experimentally observed increase in CH_3_OH selectivity as the applied voltage rises from ‐0.82 V_RHE_ to ‐0.94 V_RHE_.

**Figure 4:**
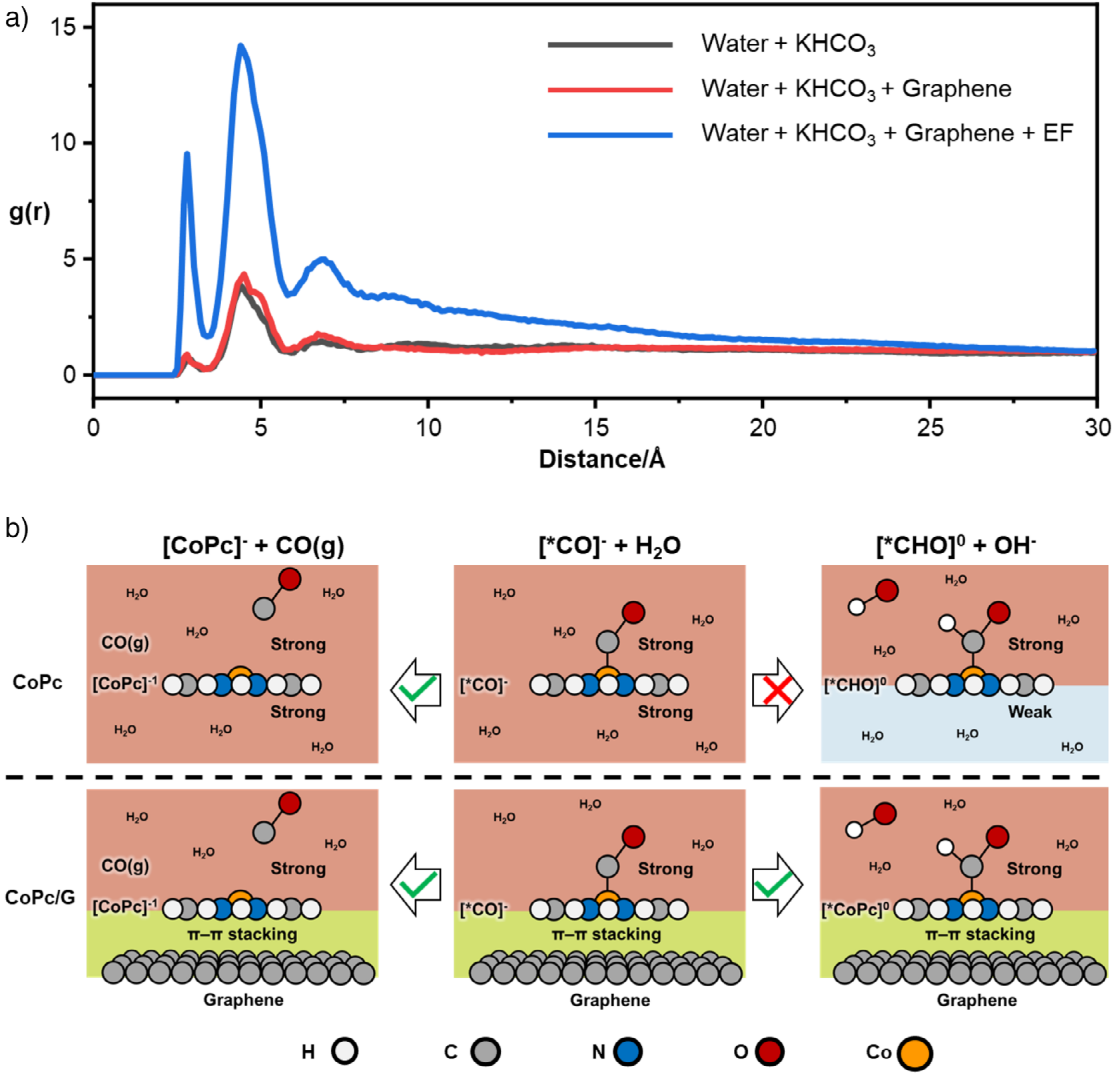
a) RDF of K^+^ around the O atom at negative species (OH^−^) in TS for *CHO formation. b) Interaction strength of CoPc with its surrounding environment in aqueous solution and when loaded onto a graphene surface.

Figure 4a shows the radial distribution functions (RDF) of K^+^ around the OH^−^ in TS for *CHO formation. In an EDL with only H_2_O and KHCO_3_, the first K^+^ peak appears around 5 Å, indicating hydrated K⁺ cannot approach OH^−^ closely enough to stabilize it, consistent with similar proton transfer energy barriers in pure water and KHCO_3_ from our FEP results. However, when the EDL includes graphene, KHCO_3_, and EF, a new K^+^ peak emerges ∼2.5 Å, suggesting partial dehydration of K⁺ to approach and stabilize OH^−^, facilitating the proton transfer step.

More importantly, among EDL components (KHCO_3_, graphene, and EF), graphene plays the most crucial role in lowering the proton transfer energy barrier. The energy of intermediates during CO desorption and protonation depends on both their intrinsic state (e.g., charge, structure) and interactions with the surrounding environment. Strong interactions stabilize the intermediate, while weak interactions increase its energy. For intermediates in the same intrinsic state, their stability is determined by the strength of environmental interactions.

Figure 4b shows that the top surface of CoPc is always exposed to water, whether in solution or immobilized on graphene; thus, top surface interactions do not drive selectivity changes. In contrast, the bottom surface behaves differently in water versus CoPc/G. During CO desorption, CoPc remains negatively charged ([CoPc]^−^), whereas during protonation of [*CO]^−^, CoPc shifts from [CoPc]^−^ to neutral ([CoPc]^0^). The solvation energies of [CoPc]^0^ and [CoPc]^−^ in water are –20.46 and –53.44 kcal/mol, respectively, with half of these values approximating the interaction strength between the bottom surface of CoPc and the surrounding water. Therefore, in solution, the bottom surface interaction remains strong (–26.72 kcal mol^−1^) during CO desorption but shifts from strong to weak (–10.23 kcal mol^−1^) during *CO protonation, weakening the intermediate‐environment interaction and disfavoring the reaction. In the CoPc/G system, the bottom surface maintains a constant π–π interaction with graphene. As a result, the interaction remains essentially unchanged during CO desorption, consistent with FEP simulations showing minimal effect of immobilization on the CO desorption barrier. For *CO protonation, the constant bottom surface interaction eliminates the unfavorable weakening seen in solution, thereby facilitating the protonation. This mechanism also explains why only π–π‐immobilized CoPc on graphene can produce CH_3_OH, while CoPc immobilized on graphene via a pyridine link can only produce CO.^[^ ^35^ ^]^

**Figure 5:**
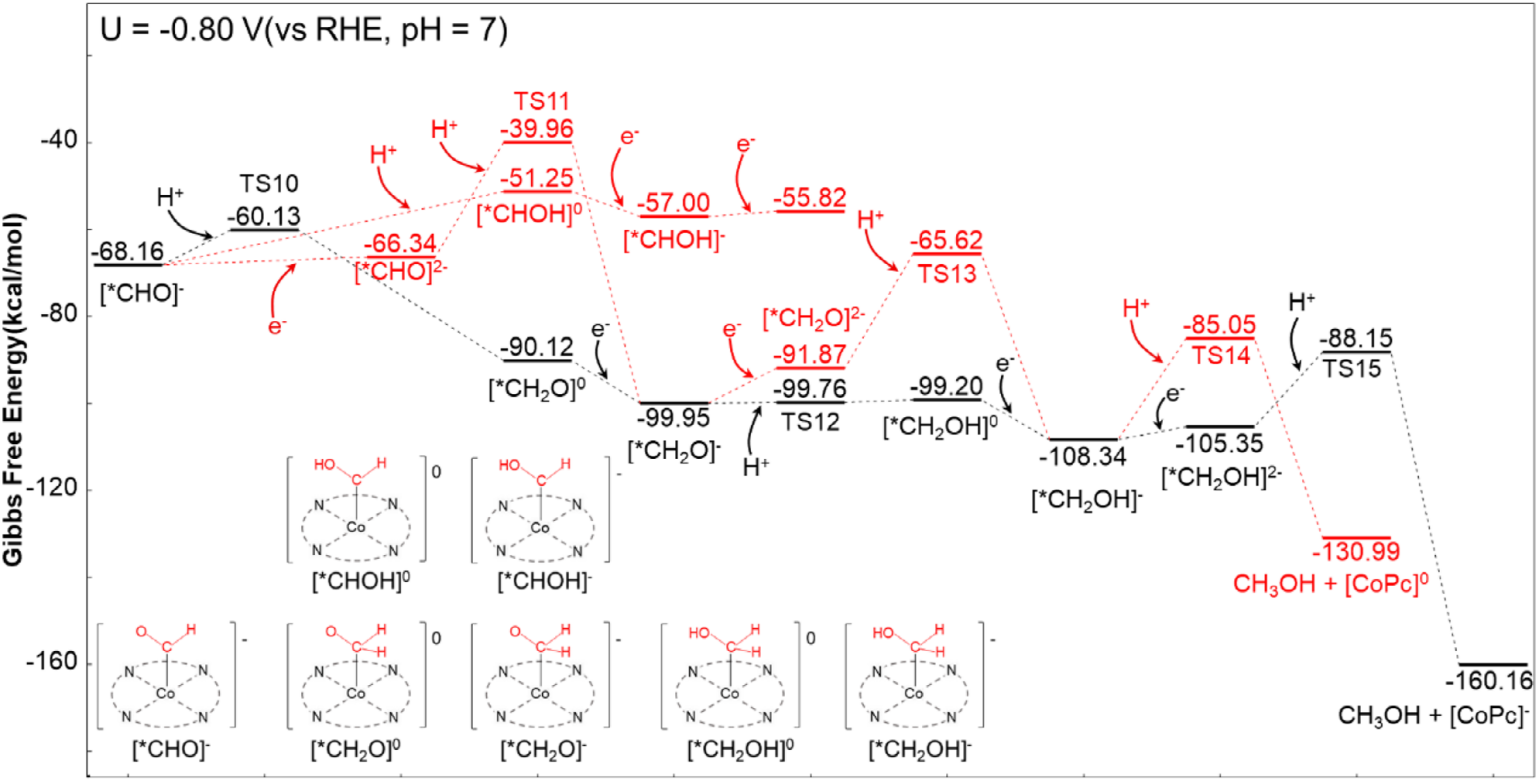
Energy profile from [*CHO]^−^ to CH_3_OH on CoPc.

After the formation of the [*CHO] intermediate, Figure 5 reveals that [*CH_2_O] acts as a key intermediate in the subsequent conversion to CH_3_OH, consistent with experimental identification of formaldehyde as an intermediate.^[^ ^36^ ^]^ The transformation from [*CH_2_OH]^2−^ to CH_3_OH is the RDS. This observation also agrees well with experimental findings that the CO‐to‐CH_3_OH conversion proceeds through a protonation step as the RDS.^[^ ^9^ ^]^ For this protonation step, based on FEP simulations, we also find that the EDL lowers the energy barrier. (Table S1)

In this study, we resolve the long‐standing puzzle of why immobilizing CoPc on a carbon support dramatically switches its electrocatalytic selectivity from CO to CH_3_OH. Through multiscale simulations, we reveal that this switch arises from profound interfacial effect. We show that the graphene support acts as a physical shield, inducing desolvation of intermediates, which counterintuitively lowers the kinetic barrier for the critical protonation step, making it competitive with facile CO desorption for the first time. This overarching environmental mechanism provides the thermodynamic foundation upon which secondary factors, such as EF and cation effects, can further promote methanol formation. This work redefines the support as an active modulator of catalytic selectivity rather than a passive anchor. By controlling interfacial environments, it offers a new design principle for tuning selectivity in CO_2_ reduction and other complex electrocatalytic reactions.

## Supporting Information

All details of the computational study, including the DFT functional, basis sets, and software packages used, as well as the force field parametrization, molecular dynamics simulations, free energy perturbation, and the corresponding geometry file.

## Conflict of Interests

The authors declare no conflict of interest.

## Supporting information



Supporting Information

## Data Availability

The data that support the findings of this study are openly available in Zenodo at https://doi.org/10.5281/zenodo.17098658, reference number 17098658.
