# Supplementary material for: Support‐Induced Interfacial Effects Steer Methanol Selectivity in CO2 Electroreduction by Immobilized Cobalt Phthalocyanine
Source: Angew Chem Int Ed Engl. 2025 Nov 22;65(8):e21683. doi: 10.1002/anie.202521683 (PMC12910136; doi:10.1002/anie.202521683)
Supplement: Supplementary file 1 — Supporting Information [file ANIE-65-e21683-s001.docx]

# Supplementary Information

**Support-Induced Interfacial Effects Steer Methanol Selectivity in CO2 Electroreduction by Immobilized Cobalt Phthalocyanine**

Ke Ye^[a]^, Min Hu^[a]^, Guozhen Zhang^[b]^, Mårten S. G. Ahlquist*^[a]^

[a] Division of Theoretical Chemistry and Biology, KTH Royal Institute of Technology, 10691Stockholm, Sweden

Email: [ahlqui@kth.se](mailto:ahlqui@kth.se)

[b] Hefei National Research Center for Physical Sciences at the Microscale, School of Chemistry and Materials Science, University of Science and Technology of China, Hefei 230026, China.

Contents

[1. Computational Detail 2](#_Toc194997398)

[1.1 DFT calculation 2](#_Toc194997399)

[1.2 MD simulation 3](#_Toc194997400)

[2. Energy profile, important structures, and FEP results 4](#_Toc194997401)

[3. Table of energies 7](#_Toc194997402)

[4. Cartesian coordinates of optimized structures 9](#_Toc194997403)

[5. Reference 67](#_Toc194997404)

## Computational Detail

An essential consideration in CoPc catalyzed CO_2_RR is the potential protonation of N-atoms in the phthalocyanine ring. Some previous computational studies suggest that these N-atoms are susceptible to protonation^[1]^; this process may lead to catalyst deactivation^[2]^. Since no experimental evidence confirms that N-protonated CoPc is the true active species, we have treated CoPc as the catalytically active species in this work.

### 1.1 DFT calculation

All DFT calculations were executed using the Gaussian16 package^[3]^. The calculations were executed using B3LYP-D3 density functional^[4]^ with solvation effects modeled via the SMD continuum approach^[5]^. For geometry optimizations, we employed a mixed basis set: the SDD^[6]^ was used for the Co atom, and all the other elements were treated with 6-31G(d,p) basis set. Vibrational frequency calculations were performed at the same theoretical level to confirm the nature of stationary point and to obtain the Gibbs free energy corrections at 298.15 K. To enhance the accuracy of electronic energies, we performed subsequent single-point calculations with an expanded basis set (6-311+G(2df,2p)) for all atoms except cobalt, which retained the SDD treatment. For electrochemical calculations, we referenced all potentials to the standard hydrogen electrode (SHE) using its established absolute potential of 4.281 V^[7]^ (corresponding to an electron affinity of 98.7 kcal/mol^[8]^). Unless otherwise noted, all the redox potentials reported in the present work are relative to RHE (pH = 7). The proton's thermodynamic parameters were incorporated using its gas-phase Gibbs free energy (-6.3 kcal/mol) and aqueous solvation free energy (-264.0 kcal/mol^[8]^), yielding a total aqueous-phase Gibbs free energy of -270.3 kcal/mol. The standard state corrections were implemented as follows: a 1.9 kcal/mol adjustment was applied to most species to account for the concentration change from ideal gas (1 atm) to aqueous solution (1 M).

### 1.2 MD simulation

All Molecular Dynamics (MD) simulations were performed using the GROMACS 2022.2 package^[9]^. Restrained electrostatic potential (RESP) charges were computed to partition the electron by Multiwfn software^[10]^. Forcefield parameters are based on the OPLS-AA force field^[11]^. The equilibrium bond lengths, angles, and dihedrals were taken from the optimized structures performed by Gaussian16. Sobtop software^[12]^ was used to help build the parameters that were still missing. The TIP/3P model was used because of its superior ability to replicate the experimental dielectric constant of water compared to other widely used water models^[13]^. To avoid self-interactions under periodic boundary conditions, a sufficiently large box of 69.1 × 68.4 × 66.5 Å^3^ was used for all simulations. We used a graphene sheet to model the interaction between the CoPc and the carbon nanotubes since large multi-walled nanotubes were used in the experiments. The diameter of the CNT is much larger than the size of a CoPc molecule, rendering the local surface on which CoPc is adsorbed effectively planar. Such an approximation has also been adopted in previous theoretical studies^[1a]^, where it was confirmed that modeling CoPc immobilized on CNTs using CoPc on graphene yields results largely insensitive to the strain effects expected for large-diameter CNTs. Following our previous work^[14]^, to accurately describe the non-covalent interactions between graphene and CoPc, we increased the epsilon value of the graphene carbon atoms, as shown below:

[ atomtypes ]

; name mass charge ptype sigma eps

ca 6 12.010736 0.000000 A 3.550000E-01 5.85760E-01
While the standard is:
[ atomtypes ]

; name mass charge ptype sigma eps

ca 6 12.010736 0.000000 A 3.550000E-01 2.92880E-01

Further details of the FEP simulation input files are available at: <https://zenodo.org/records/17098658>

#### 1.2.1 .mdp file for FEP simulation:

title = OPLS Lysozyme NPT equilibration

; Run parameters

integrator = md ; leap-frog integrator

nsteps = 2000000 ; 1 * 100000000 = 100000 ps (100 ns)

dt = 0.001 ; 1 fs

; Output control

nstxout = 100000 ; suppress bulky .trr file by specifying

nstvout = 100000 ; 0 for output frequency of nstxout,

nstfout = 100000 ; nstvout, and nstfout

nstenergy = 200 ; save energies every 10.0 ps

nstlog = 100000 ; update log file every 10.0 ps

nstxout-compressed = 200 ; save compressed coordinates every 10.0 ps

compressed-x-grps = System ; save the whole system

; Bond parameters

continuation = yes ; Restarting after NPT

constraint_algorithm = lincs ; holonomic constraints

constraints = h-bonds ; bonds involving H are constrained

lincs_iter = 1 ; accuracy of LINCS

lincs_order = 4 ; also related to accuracy

; Neighborsearching

cutoff-scheme = Verlet ; Buffered neighbor searching

ns_type = grid ; search neighboring grid cells

nstlist = 10 ; 20 fs, largely irrelevant with Verlet scheme

rcoulomb = 1.0 ; short-range electrostatic cutoff (in nm)

rvdw = 1.0 ; short-range van der Waals cutoff (in nm)

; Electrostatics

coulombtype = PME ; Particle Mesh Ewald for long-range electrostatics

pme_order = 4 ; cubic interpolation

fourierspacing = 0.16 ; grid spacing for FFT

; Temperature coupling is on

tcoupl = V-rescale ; modified Berendsen thermostat

tc-grps = system ; two coupling groups - more accurate

tau_t = 0.1 ; time constant, in ps

ref_t = 300 ; reference temperature, one for each group, in K

; Pressure coupling is on

pcoupl = Berendsen ; Pressure coupling on in NPT

pcoupltype = isotropic ; uniform scaling of box vectors

tau_p = 2.0 ; time constant, in ps

ref_p = 1.0 ; reference pressure, in bar

compressibility = 4.5e-5 ; isothermal compressibility of water, bar^-1

refcoord_scaling = com

;ref_p = 1.0 ; reference pressure, in bar

;compressibility = 4.5e-5 ; isothermal compressibility of water, bar^-1

; Periodic boundary conditions

pbc = xyz ; 3-D PBC

; Dispersion correction

DispCorr = EnerPres ; account for cut-off vdW scheme

; Velocity generation

gen_vel = no ; Velocity generation is off

;define = -DPOSRES ; position restrain the graphene

;electric-field-z = -0.5 0 0 0; Electric field on z with a value in V/nm

; Free energy control parameters

free_energy = yes

init_lambda_state = 0

delta_lambda = 0

calc_lambda_neighbors = 1

couple-lambda0 = vdw-q

couple-lambda1 = vdw-q

couple-intramol = yes

; Vectors of lambda specified here

; init_lambda_state 0 1 2 3 4 5 6 7 8 9 10

vdw_lambdas = 0.00 0.00 0.00 0.00 0.00 0.00 0.00 0.00 0.00

coul_lambdas = 0.00 .1250 .2500 .3750 .5000 .6250 .7500 .8750 1.0000

bonded_lambdas = 0.00 .1250 .2500 .3750 .5000 .6250 .7500 .8750 1.0000

restraint_lambdas = 0.00 .1250 .2500 .3750 .5000 .6250 .7500 .8750 1.0000

; Masses are not changing (particle identities are the same at lambda = 0 and lambda = 1)

mass_lambdas = 0.00 0.00 0.00 0.00 0.00 0.00 0.00 0.00 0.00

; Not doing simulated temperting here

temperature_lambdas = 0.00 0.00 0.00 0.00 0.00 0.00 0.00 0.00 0.00

; Options for the decoupling

sc-alpha = 0.5

sc-coul = no ; linear interpolation of Coulomb (none in this case)

sc-power = 1

sc-sigma = 0.3

nstdhdl = 10

disre = simple

nstdisreout = 0

## Energy profile and important structures


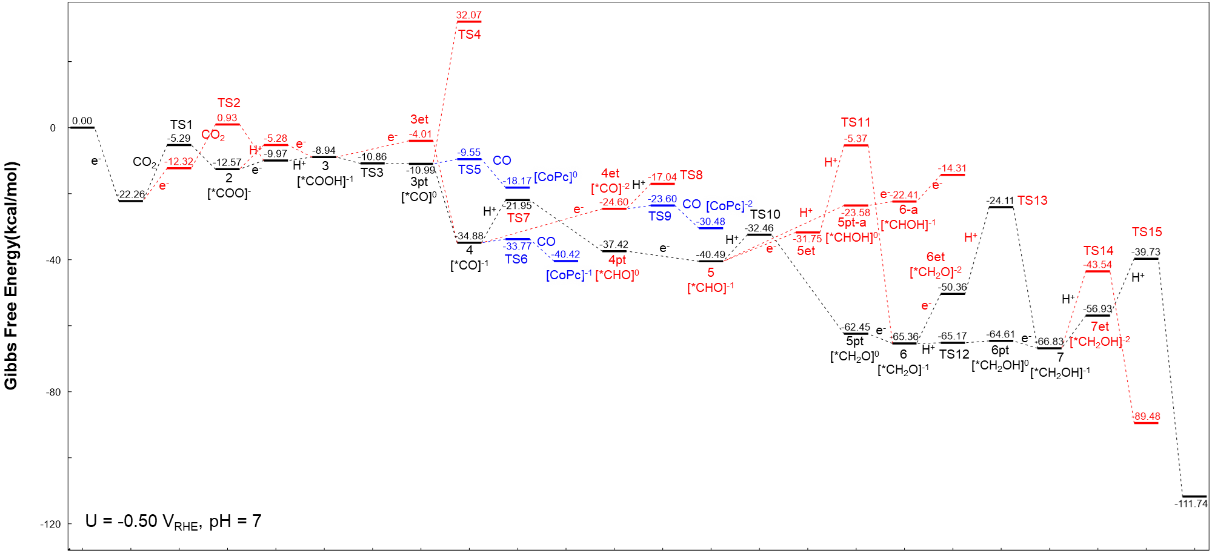


Figure S1: The free energy change of CO_2_RR to *CHO catalyzed by CoPc in water environment under applied potentials of -0.50 V_RHE_. At these low overpotentials, the formation of the [*CO]^0^ intermediate is favored. For [*CO]^0^_,_ the CO desorption barrier and protonation barrier are 1.44 and 43.06 kcal/mol, respectively.


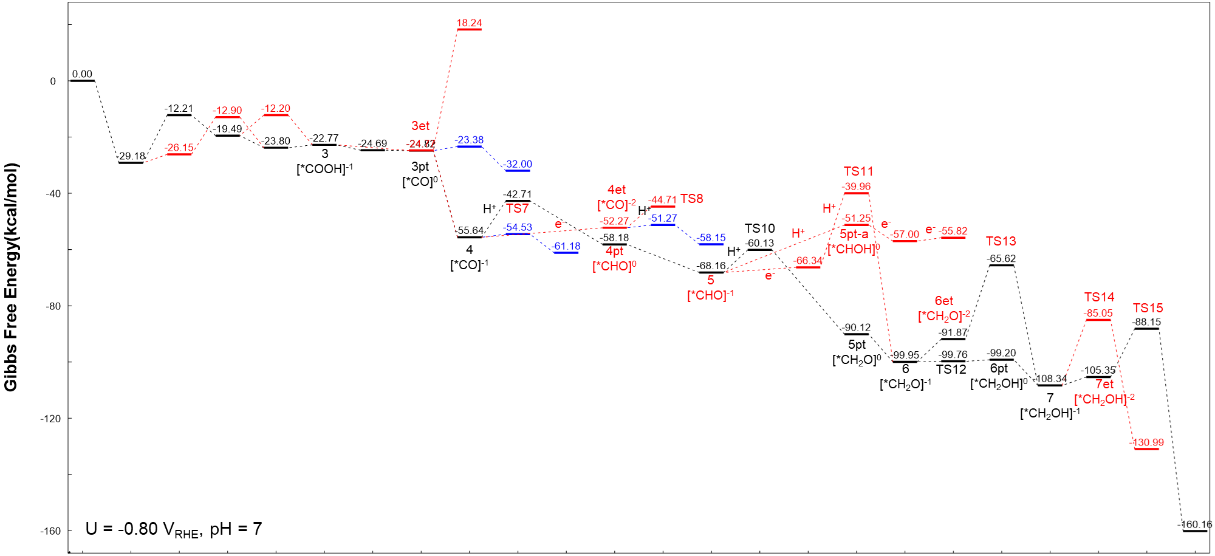


Figure S2 The free energy change of CO_2_RR to *CHO catalyzed by CoPc in water environment under applied potentials of -0.80 V_RHE_. At these overpotentials, the formation of the [*CO]^-^ intermediate is favored. For [*CO]^-^_,_ the CO desorption barrier and protonation barrier are 1.10 and 12.93 kcal/mol, respectively.


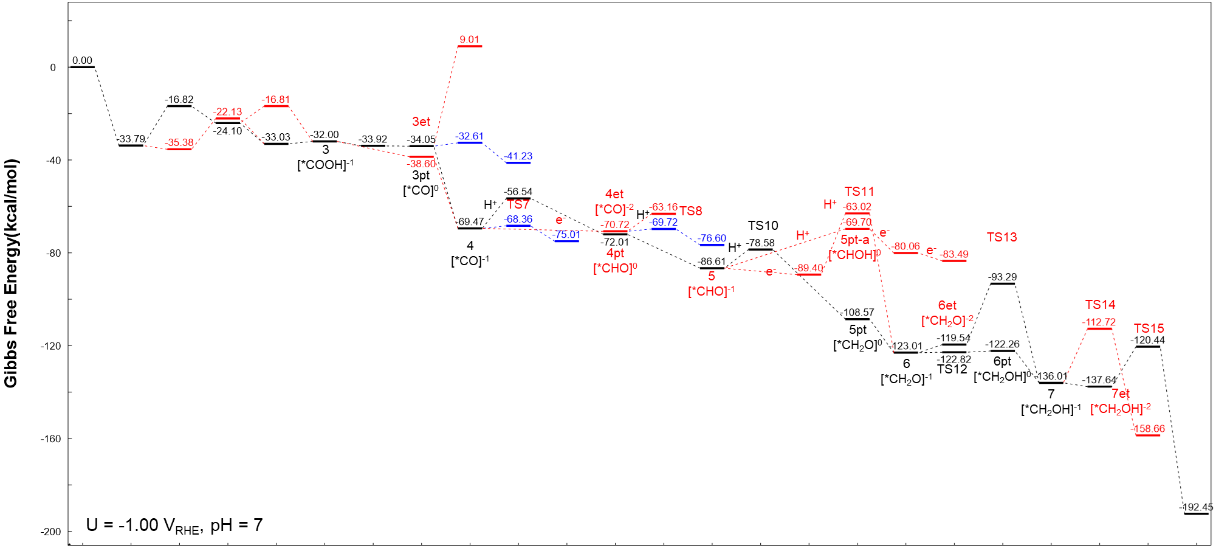


Figure S3 The free energy change of CO_2_RR to *CHO catalyzed by CoPc in water environment under applied potentials of -1.00 V_RHE_. At these high overpotentials, the formation of the [*CO]^2-^ intermediate is favored. For [*CO]^2-^_,_ the CO desorption barrier and protonation barrier are 1.00 and 7.56 kcal/mol, respectively.

From *CHO to CH_3_OH, our calculations identified *CH_2_O as a key intermediate, consistent with experimental observations of formaldehyde as an intermediate^[15]^. We also found that the conversion of *CHO to CH_3_OH requires a specific overpotential, with the rate-determining step being the proton transfer process. This aligns with experimental findings that the conversion of CO to CH_3_OH demands a relatively high overpotential (U <-0.77 V_RHE_)^[2]^, and the RDS from *CO to CH_3_OH is a protonation step.


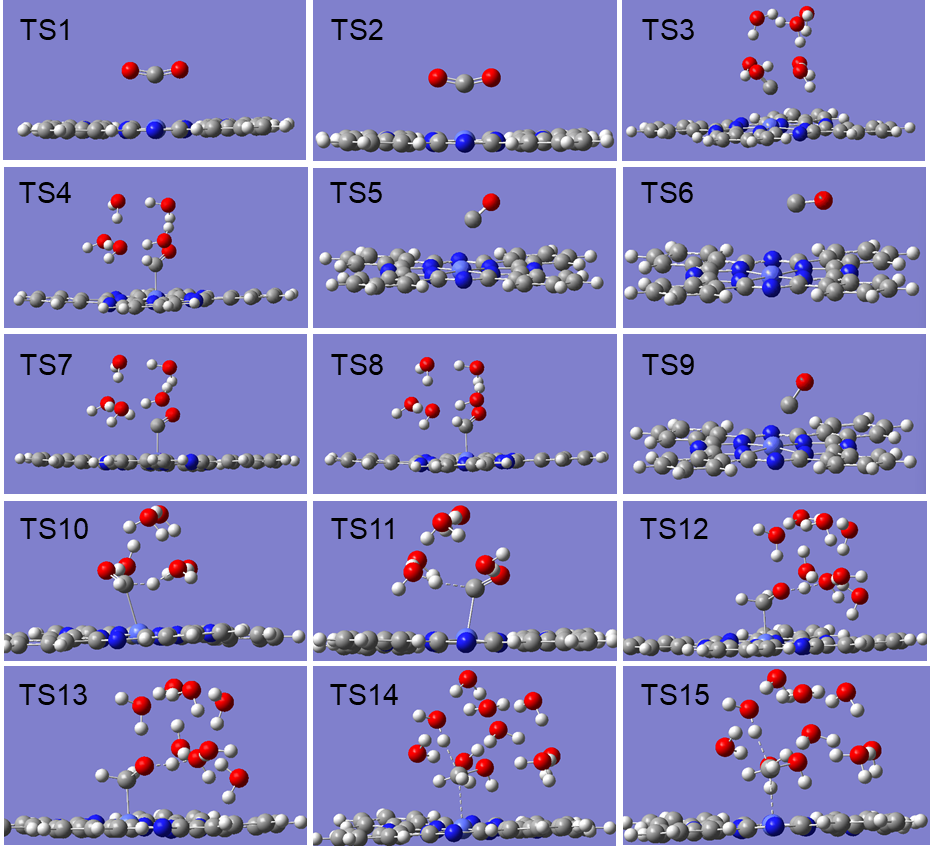


Figure S4: Transition state structures involved in CO and CH₃OH formation during CO₂RR catalyzed by CoPc.


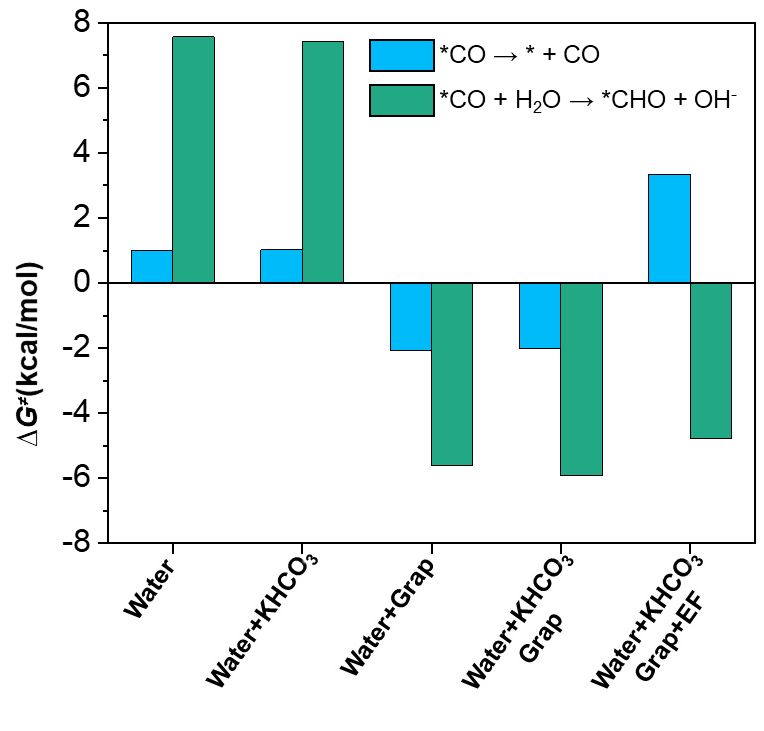


Figure S5 Energy barriers for CO desorption and protonation of the [*CO]^2-^ in different EDL environments. Compared to pure aqueous solution, the CO desorption barrier increases slightly, while the protonation barrier decreases significantly, indicating that the initial reaction selectivity in the EDL favors CH_3_OH formation.

Table S1. FEP results for the energy barriers of key protonation processes (in kcal/mol).

|  | water | CoPc/G in **KHCO_3_** solution with EF | CoPc/G in **LiHCO_3_** solution with EF |
| --- | --- | --- | --- |
| [*CO]^-^ →[*CHO]^0^ | 12.93 | 0.88 | 0.23 |
| [*CH_2_OH]^2-^ → CH_3_OH | 17.19 | 8.55 | 7.24 |

## Table of energies

Table S2. Calculated energies in Hartree. (The symbol “/” in the table indicates either unconverged or physically unreasonable structures.)

| Intermediates | spin | opt | Thermal correction to Gibbs Free Energy | Single point calculation with large basis set |
| --- | --- | --- | --- | --- |
| 0 | Doublet | -1813.25253 | 0.356943 | -1813.755919 |
|  | Quartet | -1813.2525 | 0.351863 | -1813.757366 |
|  | Sextet | -1813.2364 | 0.346854 | -1813.740907 |
| 1 | Singlet | -1813.408748 | 0.358551 | -1813.920066 |
|  | Triplet | -1813.408169 | 0.356645 | -1813.921495 |
|  | Quintet | -1813.354574 | 0.353141 | -1813.867486 |
| 1et | Doublet | -1813.506521 | 0.355557 | -1814.02844 |
|  | Quartet | -1813.500976 | 0.354341 | -1814.02491 |
|  | Sextet | -1813.408905 | 0.346713 | -1813.933444 |
| TS1 | Singlet | -2001.985291 | 0.364737 | -2002.569387 |
|  | Triplet | / |  |  |
|  | Quintet | / |  |  |
| TS2 | Doublet | -2002.083957 | 0.360023 | -2002.678651 |
|  | Quartet | / |  |  |
|  | Sextet | / |  |  |
| 2 | Singlet | -2001.996647 | 0.367178 | -2002.583429 |
|  | Triplet | -2001.951781 | 0.359902 | -2002.539694 |
|  | Quintet | -2001.937277 | 0.355901 | -2002.527706 |
| 2et | Doublet | -2002.102901 | 0.362533 | -2002.698526 |
|  | Quartet | -2002.057614 | 0.362296 | -2002.654346 |
|  | Sextet | -2002.034668 | 0.358176 | -2002.627633 |
| 2pt | Singlet | -2002.455376 | 0.380543 | -2003.031153 |
|  | Triplet | -2002.415079 | 0.377036 | -2002.991818 |
|  | Quintet | / |  |  |
| 3 | Doublet | -2002.574028 | 0.378319 | -2003.15863 |
|  | Quartet | -2002.530223 | 0.373726 | -2003.115326 |
|  | Sextet | / |  |  |
| TS3 | Doublet | -2384.814305 | 0.482541 | -2385.577043 |
|  | Quartet | / |  |  |
|  | Sextet | / |  |  |
| 3et | Singlet | -2002.675661 | 0.375632 | -2003.271413 |
|  | Triplet | -2002.6737 | 0.372287 | -2003.26864 |
|  | Quintet | -2002.671281 | 0.369495 | -2003.265504 |
| 3pt | Doublet | -1926.557462 | 0.358734 | -1927.108456 |
|  | Quartet | -1926.5667 | 0.355352 | -1927.113183 |
|  | Sextet | -1926.547826 | 0.356728 | -1927.093813 |
| TS4 | Doublet | -2308.778022 | 0.468969 | -2309.498233 |
|  | Quartet | / |  |  |
|  | Sextet | / |  |  |
| TS5 | Doublet | / |  |  |
|  | Quartet | -1926.564632 | 0.356127 | -1927.111661 |
|  | Sextet | / |  |  |
| 4 | Singlet | -1926.714439 | 0.360134 | -1927.265999 |
|  | Triplet | -1926.722429 | 0.357309 | -1927.2771 |
|  | Quintet | -1926.677681 | 0.35453 | -1927.232898 |
| TS6 | Singlet | / |  |  |
|  | Triplet | -1926.720247 | 0.357425 | -1927.27546 |
|  | Quintet | / |  |  |
| TS7 | Singlet | -2308.948397 | 0.470099 | -2309.675599 |
|  | Triplet | / |  |  |
|  | Quintet | / |  |  |
| 4et | Doublet | -1926.817749 | 0.356046 | -1927.383341 |
|  | Quartet | -1926.816748 | 0.354944 | -1927.38179 |
|  | Sextet | / |  |  |
| TS8 | Doublet | -2309.056735 | 0.466527 | -2309.792773 |
|  | Quartet | -2309.010374 | 0.463809 | -2309.747347 |
|  | Sextet | / |  |  |
| TS9 | Doublet | -1926.815751 | 0.355607 | -1927.381304 |
|  | Quartet | / |  |  |
|  | Sextet | / |  |  |
| 4pt | Singlet | -1927.200121 | 0.376294 | -1927.746111 |
|  | Triplet | -1927.158071 | 0.371175 | -1927.704749 |
|  | Quintet | -1927.115129 | 0.363792 | -1927.661112 |
| 5 | Doublet | -1927.315312 | 0.371723 | -1927.8703 |
|  | Quartet | -1927.271133 | 0.368769 | -1927.826773 |
|  | Sextet | -1927.247038 | 0.363474 | -1927.800263 |
| TS10 | Doublet | -2309.48104 | 0.487442 | -2310.265756 |
|  | Quartet | -2309.498452 | 0.475016 | -2310.224378 |
|  | Sextet | / |  |  |
| 5et | Singlet | -1927.413186 | 0.370661 | -1927.979191 |
|  | Triplet | -1927.411572 | 0.368794 | -1927.977089 |
|  | Quintet | -1925.411572 | 0.36021 | -1927.916776 |
| 5pt | Doublet | -1927.815302 | 0.383308 | -1928.362852 |
|  | Quartet | -1927.77241 | 0.37928 | -1928.320758 |
|  | Sextet | -1927.756018 | 0.373224 | -1928.30399 |
| 5pt-a  5pt-a | Doublet | -1927.758458 | 0.385643 | -1928.303242 |
|  | Quartet | -1927.716593 | 0.380846 | -1928.261807 |
|  | Sextet | -1927.705011 | 0.377828 | -1928.24944 |
| TS11 | Singlet | -2309.623852 | 0.479658 | -2310.361195 |
|  | Triplet | -2309.568926 | 0.483154 |  |
|  | Quintet | / |  |  |
| 6 | Singlet | -1927.920816 | 0.38036 | -1928.476054 |
|  | Triplet | -1927.928045 | 0.376721 | -1928.484778 |
|  | Quintet | / |  |  |
| 6-a | Singlet | -1927.845268 | 0.382469 | -1928.403065 |
|  | Triplet | -1927.866072 | 0.380949 | -1928.420555 |
|  | Quintet | -1927.820651 | 0.379579 | -1928.375829 |
| 6et | Doublet | -1928.020552 | 0.380072 | -1928.588095 |
|  | Quartet | -1928.012325 | 0.376112 | -1928.579215 |
|  | Sextet | -1927.966474 | 0.375701 | -1928.527775 |
| TS12 | Singlet | -2539.531864 | 0.564018 | -2540.360097 |
|  | Triplet | / |  |  |
|  | Quintet | / |  |  |
| TS13 | Doublet | / |  |  |
|  | Quartet | -2539.596949 | 0.557229 | -2540.434459 |
|  | Sextet | / |  |  |
| 6pt | Singlet | -1928.408553 | 0.40207 | -1928.954895 |
|  | Triplet | -1928.365372 | 0.394522 | -1928.912469 |
|  | Quintet | / |  |  |
| 7 | Doublet | -1928.520719 | 0.396071 | -1929.076305 |
|  | Quartet | -1928.475822 | 0.393922 | -1929.03213 |
|  | Sextet | -1928.446269 | 0.387127 | -1929.004036 |
| 7et | Singlet | -1928.616014 | 0.394444 | -1929.182777 |
|  | Triplet | -1928.614863 | 0.394154 | -1929.181144 |
|  | Quintet | -1928.586165 | 0.38717 | -1929.153723 |
| TS14 | Doublet | -2616.528368 | 0.593031 | -2617.401256 |
|  | Quartet | / |  |  |
|  | Sextet | / |  |  |
| TS15 | Singlet | -2616.617891 | 0.592149 | -2617.498352 |
|  | Triplet | -2616.63855 | 0.59055 | -2617.520803 |
|  | Quintet | / |  |  |

## Cartesian coordinates of optimized structures

(The first number before the coordinates represents the charge, and the second indicates the spin.)

**0**

0 4

N 1.385339 -1.361442 0.000231

C 2.730672 -1.149459 0.000409

C 1.170905 -2.745848 0.000247

C 3.437491 -2.430404 0.000550

C 2.449982 -3.432535 0.000448

N 1.376194 1.370630 0.000145

C 2.722903 1.167388 0.000265

C 1.152792 2.753596 0.000020

C 2.427369 3.448605 0.000065

C 3.421341 2.452879 0.000213

N -1.385339 1.361442 -0.000220

C -1.170906 2.745848 -0.000175

C -2.730672 1.149459 -0.000323

C -2.449982 3.432535 -0.000201

C -3.437491 2.430404 -0.000284

N -1.376194 -1.370630 -0.000153

C -1.152792 -2.753596 -0.000115

C -2.722903 -1.167388 -0.000328

C -3.421341 -2.452879 -0.000472

C -2.427368 -3.448605 -0.000319

C 4.125275 5.129539 0.000034

H 4.421839 6.173644 -0.000040

C 5.116210 4.134203 0.000181

C 2.766416 4.801263 -0.000023

H 2.003798 5.572646 -0.000142

C 4.774019 2.777464 0.000275

H 5.537029 2.006514 0.000389

H 6.162076 4.423554 0.000221

C 5.143087 -4.100880 0.000805

H 6.190805 -4.383469 0.000942

C 4.158584 -5.102570 0.000703

C 4.792220 -2.746353 0.000726

H 5.550330 -1.970582 0.000798

C 2.797654 -4.782982 0.000523

H 2.039969 -5.559207 0.000438

H 4.461849 -6.144744 0.000763

C -5.116210 -4.134203 -0.000730

H -6.162076 -4.423554 -0.000883

C -4.125274 -5.129539 -0.000577

C -4.774019 -2.777464 -0.000685

H -5.537029 -2.006514 -0.000793

C -2.766416 -4.801263 -0.000372

H -2.003798 -5.572646 -0.000253

H -4.421839 -6.173644 -0.000619

C -4.158584 5.102570 -0.000174

H -4.461848 6.144744 -0.000132

C -5.143087 4.100880 -0.000255

C -2.797654 4.782982 -0.000140

H -2.039969 5.559207 -0.000073

C -4.792220 2.746353 -0.000303

H -5.550330 1.970583 -0.000367

H -6.190805 4.383470 -0.000276

N -0.011155 3.377763 -0.000093

N 0.011155 -3.377763 0.000076

N -3.387171 -0.011158 -0.000396

N 3.387171 0.011158 0.000415

Co -0.000000 0.000000 0.000002

**1**

-1 3

N -0.000055 -1.949401 -0.000013

C -1.115719 -2.762311 -0.000028

C 1.115567 -2.762375 -0.000010

C -0.710061 -4.140566 -0.000035

C 0.709832 -4.140606 -0.000022

N -1.947814 0.000053 -0.000004

C -2.762898 -1.120127 -0.000022

C -2.762837 1.120281 0.000006

C -4.164555 0.702404 -0.000005

C -4.164594 -0.702172 -0.000024

N 0.000055 1.949401 0.000013

C -1.115567 2.762375 0.000023

C 1.115719 2.762311 0.000017

C -0.709832 4.140606 0.000035

C 0.710061 4.140566 0.000030

N 1.947814 -0.000053 0.000004

C 2.762837 -1.120281 0.000005

C 2.762898 1.120127 0.000009

C 4.164594 0.702172 0.000012

C 4.164555 -0.702404 0.000011

C -6.555079 0.703350 -0.000017

H -7.501283 1.235725 -0.000014

C -6.555119 -0.702986 -0.000036

C -5.358509 1.423264 -0.000001

H -5.357922 2.508443 0.000014

C -5.358588 -1.422967 -0.000040

H -5.358062 -2.508145 -0.000055

H -7.501352 -1.235308 -0.000048

C -0.708618 -6.537158 -0.000053

H -1.238102 -7.485306 -0.000065

C 0.708250 -6.537199 -0.000040

C -1.426358 -5.348481 -0.000051

H -2.512035 -5.351612 -0.000061

C 1.426059 -5.348563 -0.000025

H 2.511735 -5.351759 -0.000016

H 1.237680 -7.485377 -0.000043

C 6.555118 0.702986 0.000021

H 7.501352 1.235309 0.000024

C 6.555079 -0.703350 0.000020

C 5.358588 1.422967 0.000017

H 5.358062 2.508145 0.000018

C 5.358509 -1.423264 0.000015

H 5.357922 -2.508443 0.000015

H 7.501283 -1.235725 0.000023

C -0.708250 6.537199 0.000055

H -1.237680 7.485377 0.000065

C 0.708618 6.537158 0.000051

C -1.426058 5.348563 0.000047

H -2.511735 5.351759 0.000051

C 1.426358 5.348480 0.000038

H 2.512035 5.351611 0.000034

H 1.238103 7.485306 0.000057

N -2.405624 2.381011 0.000020

N 2.405624 -2.381011 0.000000

N 2.405756 2.380878 0.000013

N -2.405756 -2.380878 -0.000034

Co 0.000000 0.000000 0.000000

**TS1**

-1 1

N 1.356467 1.364205 -0.233507

C 1.154474 2.728861 -0.222370

C 2.720656 1.159100 -0.203561

C 2.430712 3.429514 -0.199060

C 3.423699 2.434138 -0.184923

N -1.358173 1.362523 -0.233562

C -1.157871 2.727426 -0.222447

C -2.722105 1.155728 -0.203644

C -3.426730 2.429894 -0.185094

C -2.434976 3.426499 -0.199224

N -1.356473 -1.364210 -0.233524

C -2.720664 -1.159108 -0.203586

C -1.154482 -2.728868 -0.222451

C -3.423706 -2.434146 -0.184997

C -2.430719 -3.429521 -0.199169

N 1.358160 -1.362528 -0.233597

C 2.722092 -1.155735 -0.203662

C 1.157858 -2.727432 -0.222524

C 2.434962 -3.426506 -0.199293

C 3.426716 -2.429902 -0.185125

C -5.120216 4.115206 -0.143270

H -6.165723 4.406786 -0.121064

C -4.127216 5.112914 -0.157422

C -4.782430 2.761533 -0.157067

H -5.548082 1.992789 -0.145430

C -2.772314 4.780970 -0.185266

H -2.007011 5.550090 -0.195449

H -4.423414 6.157283 -0.146027

C 4.120864 5.118018 -0.157118

H 4.415766 6.162753 -0.145669

C 5.115098 4.121539 -0.142958

C 2.766375 4.784400 -0.185038

H 2.000123 5.552575 -0.195228

C 4.778986 2.767451 -0.156824

H 5.545586 1.999651 -0.145184

H 6.160241 4.414415 -0.120692

C 4.127200 -5.112923 -0.157467

H 4.423397 -6.157292 -0.146072

C 5.120201 -4.115215 -0.143281

C 2.772299 -4.780977 -0.185341

H 2.006996 -5.550097 -0.195547

C 4.782416 -2.761542 -0.157072

H 5.548068 -1.992799 -0.145405

H 6.165707 -4.406796 -0.121051

C -5.115104 -4.121550 -0.143095

H -6.160247 -4.414427 -0.120841

C -4.120870 -5.118028 -0.157286

C -4.778994 -2.767461 -0.156915

H -5.545594 -1.999662 -0.145250

C -2.766381 -4.784409 -0.185193

H -2.000129 -5.552583 -0.195405

H -4.415772 -6.162764 -0.145869

N -3.360344 -0.002088 -0.190857

N 3.360334 0.002079 -0.190851

N 0.002086 -3.370041 -0.221027

N -0.002096 3.370034 -0.220926

Co -0.000004 -0.000001 -0.212398

C 0.000064 0.000039 2.363115

O 1.166708 0.000227 2.572575

O -1.166571 -0.000120 2.572632

**1et**

-2 2

N 0.015851 -1.951787 0.000016

C 1.137619 -2.760967 0.000037

C -1.094295 -2.778350 0.000010

C 0.747586 -4.129271 0.000032

C -0.683138 -4.140216 0.000023

N 1.929942 0.014412 0.000007

C 2.769362 -1.105669 0.000030

C 2.752000 1.148457 -0.000005

C 4.157591 0.733664 0.000011

C 4.168725 -0.668894 0.000034

N -0.015851 1.951787 -0.000018

C 1.094295 2.778350 -0.000025

C -1.137619 2.760968 -0.000016

C 0.683138 4.140216 -0.000040

C -0.747586 4.129271 -0.000038

N -1.929942 -0.014412 -0.000007

C -2.752000 -1.148457 -0.000008

C -2.769362 1.105669 -0.000010

C -4.168725 0.668894 -0.000009

C -4.157591 -0.733664 -0.000008

C 6.551546 0.753674 0.000027

H 7.492964 1.294905 0.000024

C 6.562637 -0.651255 0.000051

C 5.346668 1.462426 0.000007

H 5.336472 2.547791 -0.000013

C 5.368956 -1.378798 0.000053

H 5.375881 -2.464200 0.000070

H 7.512415 -1.177646 0.000068

C 0.762921 -6.533972 0.000035

H 1.298930 -7.479023 0.000039

C -0.660671 -6.544951 0.000025

C 1.468047 -5.342309 0.000039

H 2.554581 -5.339362 0.000048

C -1.384452 -5.364579 0.000022

H -2.470900 -5.379019 0.000012

H -1.181985 -7.498208 0.000020

C -6.562637 0.651256 -0.000006

H -7.512415 1.177647 -0.000004

C -6.551546 -0.753674 -0.000006

C -5.368956 1.378798 -0.000008

H -5.375881 2.464200 -0.000009

C -5.346668 -1.462426 -0.000007

H -5.336472 -2.547791 -0.000008

H -7.492964 -1.294905 -0.000005

C 0.660671 6.544951 -0.000068

H 1.181985 7.498209 -0.000078

C -0.762920 6.533972 -0.000064

C 1.384452 5.364580 -0.000055

H 2.470900 5.379019 -0.000055

C -1.468047 5.342309 -0.000053

H -2.554581 5.339362 -0.000047

H -1.298930 7.479023 -0.000072

N 2.400661 2.399408 -0.000022

N -2.400661 -2.399408 -0.000002

N -2.438802 2.362492 -0.000012

N 2.438803 -2.362492 0.000046

Co 0.000000 -0.000000 -0.000004

**TS2**

-2 2

N 1.505873 1.191891 -0.228534

C 1.469780 2.585568 -0.222261

C 2.850732 0.819699 -0.188698

C 2.833136 3.119367 -0.195632

C 3.696689 2.014136 -0.170203

N -1.198155 1.533445 -0.233701

C -0.831242 2.864153 -0.230741

C -2.578332 1.486310 -0.202665

C -3.114364 2.812637 -0.192210

C -1.996210 3.694679 -0.212251

N -1.505885 -1.191687 -0.231190

C -2.850713 -0.819515 -0.190691

C -1.469734 -2.585365 -0.222219

C -3.696655 -2.013928 -0.170837

C -2.833077 -3.119168 -0.194940

N 1.198218 -1.533234 -0.231438

C 2.578422 -1.486079 -0.201915

C 0.831308 -2.863933 -0.228193

C 1.996286 -3.694455 -0.210411

C 3.114463 -2.812413 -0.191857

C -4.597706 4.700680 -0.158526

H -5.600377 5.118188 -0.137597

C -3.482749 5.579572 -0.177562

C -4.425245 3.324308 -0.165806

H -5.281451 2.656153 -0.150650

C -2.185542 5.088917 -0.204228

H -1.334835 5.764105 -0.218263

H -3.654024 6.652256 -0.170904

C 4.716922 4.594481 -0.150594

H 5.133335 5.597295 -0.141989

C 5.581977 3.487480 -0.125769

C 3.330082 4.421999 -0.185791

H 2.661049 5.276436 -0.204320

C 5.080453 2.182746 -0.135603

H 5.747805 1.327021 -0.115997

H 6.655288 3.650076 -0.098397

C 3.482901 -5.579355 -0.179622

H 3.654199 -6.652042 -0.174159

C 4.597874 -4.700464 -0.161648

C 2.185647 -5.088695 -0.203877

H 1.334928 -5.763884 -0.217072

C 4.425384 -3.324086 -0.167739

H 5.281605 -2.655934 -0.153377

H 5.600580 -5.117973 -0.142496

C -5.581965 -3.487249 -0.126741

H -6.655294 -3.649836 -0.100036

C -4.716889 -4.594262 -0.150353

C -5.080444 -2.182521 -0.137098

H -5.747813 -1.326785 -0.118538

C -3.330029 -4.421797 -0.184783

H -2.660979 -5.276240 -0.202410

H -5.133310 -5.597072 -0.141590

N -3.356277 0.382871 -0.178604

N 3.356385 -0.382661 -0.177196

N -0.423493 -3.364989 -0.226395

N 0.423557 3.365210 -0.228710

Co 0.000001 0.000031 -0.192771

C -0.000219 -0.001193 2.317220

O 1.151169 -0.170463 2.562943

O -1.152161 0.167705 2.560615

**2**

-1 1

N 1.360076 1.374795 -0.186359

C 1.154091 2.734607 -0.167265

C 2.719513 1.162170 -0.192997

C 2.431631 3.433577 -0.173521

C 3.423526 2.437105 -0.192025

N -1.363066 1.371834 -0.186677

C -1.160041 2.732082 -0.167635

C -2.722036 1.156250 -0.193401

C -3.428820 2.429657 -0.192769

C -2.439092 3.428282 -0.174240

N -1.360071 -1.374793 -0.186397

C -2.719508 -1.162168 -0.193047

C -1.154089 -2.734609 -0.167324

C -3.423522 -2.437101 -0.192107

C -2.431629 -3.433575 -0.173610

N 1.363066 -1.371832 -0.186684

C 2.722035 -1.156248 -0.193395

C 1.160043 -2.732084 -0.167671

C 2.439094 -3.428280 -0.174283

C 3.428820 -2.429653 -0.192786

C -5.124527 4.109977 -0.198308

H -6.170447 4.400164 -0.206744

C -4.133481 5.109690 -0.179173

C -4.785346 2.756962 -0.206391

H -5.549071 1.986705 -0.221461

C -2.777632 4.782056 -0.168126

H -2.013907 5.552337 -0.153898

H -4.432666 6.153075 -0.172975

C 4.122383 5.118646 -0.177941

H 4.419306 6.162677 -0.171546

C 5.115595 4.121085 -0.197048

C 2.767244 4.788081 -0.167175

H 2.001853 5.556709 -0.152978

C 4.779343 2.767341 -0.205386

H 5.544729 1.998736 -0.220446

H 6.160883 4.413541 -0.205266

C 4.133486 -5.109686 -0.179242

H 4.432672 -6.153071 -0.173064

C 5.124530 -4.109972 -0.198350

C 2.777636 -4.782054 -0.168197

H 2.013913 -5.552337 -0.153989

C 4.785348 -2.756957 -0.206407

H 5.549070 -1.986698 -0.221457

H 6.170451 -4.400156 -0.206786

C -5.115594 -4.121079 -0.197180

H -6.160882 -4.413533 -0.205416

C -4.122383 -5.118642 -0.178080

C -4.779339 -2.767335 -0.205490

H -5.544724 -1.998728 -0.220545

C -2.767244 -4.788080 -0.167292

H -2.001855 -5.556709 -0.153100

H -4.419308 -6.162672 -0.171707

N -3.357237 -0.003654 -0.199029

N 3.357240 0.003656 -0.198994

N 0.003677 -3.374637 -0.151009

N -0.003675 3.374638 -0.150951

Co 0.000000 -0.000002 -0.062497

C -0.000011 -0.000010 1.919808

O 1.134532 0.000047 2.421871

O -1.134561 -0.000074 2.421858

**2et**

-2 2

N 1.359674 1.386338 -0.185500

C 1.157275 2.737579 -0.166313

C 2.739137 1.164187 -0.193664

C 2.436250 3.431322 -0.177220

C 3.433223 2.432209 -0.195358

N -1.360659 1.385364 -0.186222

C -1.159216 2.736714 -0.167201

C -2.739942 1.162233 -0.194691

C -3.434885 2.429687 -0.197252

C -2.438575 3.429537 -0.179036

N -1.359671 -1.386334 -0.185548

C -2.739135 -1.164183 -0.193722

C -1.157273 -2.737575 -0.166386

C -3.433220 -2.432205 -0.195443

C -2.436248 -3.431318 -0.177315

N 1.360663 -1.385360 -0.186231

C 2.739946 -1.162229 -0.194680

C 1.159219 -2.736710 -0.167247

C 2.438579 -3.429533 -0.179084

C 3.434889 -2.429683 -0.197264

C -5.123696 4.127384 -0.210934

H -6.168691 4.422831 -0.222540

C -4.126493 5.125691 -0.191303

C -4.792335 2.774991 -0.215395

H -5.563235 2.011069 -0.230779

C -2.775107 4.786010 -0.176457

H -2.005806 5.551588 -0.162286

H -4.419989 6.170965 -0.188164

C 4.123018 5.128608 -0.188073

H 4.415794 6.174080 -0.184393

C 5.120886 4.131016 -0.207628

C 2.771831 4.788000 -0.173997

H 2.002011 5.553053 -0.159914

C 4.790422 2.778383 -0.212775

H 5.561823 2.014969 -0.228115

H 6.165686 4.427166 -0.218635

C 4.126497 -5.125687 -0.191374

H 4.419992 -6.170961 -0.188259

C 5.123700 -4.127380 -0.210969

C 2.775110 -4.786006 -0.176535

H 2.005809 -5.551585 -0.162393

C 4.792339 -2.774987 -0.215400

H 5.563239 -2.011065 -0.230756

H 6.168694 -4.422827 -0.222569

C -5.120883 -4.131012 -0.207750

H -6.165683 -4.427162 -0.218767

C -4.123015 -5.128604 -0.188204

C -4.790419 -2.778379 -0.212874

H -5.561820 -2.014964 -0.228205

C -2.771828 -4.787996 -0.174115

H -2.002008 -5.553049 -0.160039

H -4.415792 -6.174076 -0.184541

N -3.365487 -0.001270 -0.200120

N 3.365491 0.001275 -0.200078

N 0.001148 -3.397448 -0.145345

N -0.001146 3.397452 -0.145271

Co 0.000000 -0.000000 -0.034812

C -0.000015 -0.000020 1.919666

O 1.129871 -0.002504 2.451661

O -1.129910 0.002450 2.451645

**2pt**
0 1

N -1.180169 -1.526882 -0.218711

C -0.805198 -2.851457 -0.148458

C -2.556647 -1.483587 -0.232466

C -1.986278 -3.698999 -0.140647

C -3.093494 -2.834569 -0.197708

N 1.528943 -1.187012 -0.169400

C 1.490119 -2.565747 -0.143169

C 2.858050 -0.810584 -0.211795

C 3.704522 -1.989913 -0.230755

C 2.840789 -3.098462 -0.180602

N 1.190201 1.527104 -0.157510

C 2.570187 1.489227 -0.183353

C 0.813447 2.853442 -0.123766

C 3.102253 2.840513 -0.170347

C 1.992140 3.702381 -0.126563

N -1.518911 1.192807 -0.243483

C -2.842398 0.812970 -0.260729

C -1.481032 2.568127 -0.181113

C -2.834140 3.101216 -0.197703

C -3.694805 1.991300 -0.252166

C 5.585508 -3.455167 -0.282489

H 6.657458 -3.620375 -0.321217

C 4.720338 -4.566041 -0.233354

C 5.090328 -2.152407 -0.282656

H 5.755913 -1.297037 -0.321293

C 3.337041 -4.403618 -0.183360

H 2.670834 -5.258575 -0.146725

H 5.141945 -5.566087 -0.234944

C -3.453817 -5.578512 -0.101638

H -3.620186 -6.650308 -0.063581

C -4.562965 -4.712687 -0.158103

C -2.150095 -5.084075 -0.093447

H -1.295506 -5.750471 -0.050443

C -4.398463 -3.328966 -0.208238

H -5.252381 -2.661797 -0.251912

H -5.563494 -5.133128 -0.162347

C -4.717913 4.563705 -0.194103

H -5.141850 5.562514 -0.171113

C -5.580198 3.451880 -0.248067

C -3.332714 4.404164 -0.168849

H -2.668360 5.260344 -0.126707

C -5.080903 2.150108 -0.278484

H -5.744846 1.293516 -0.318979

H -6.653166 3.614131 -0.265540

C 4.566101 4.723294 -0.172518

H 5.565171 5.146877 -0.189156

C 3.453989 5.586969 -0.129315

C 4.406120 3.338883 -0.195116

H 5.261929 2.673649 -0.230282

C 2.152304 5.089152 -0.108052

H 1.295675 5.753562 -0.077070

H 3.617375 6.659765 -0.113138

N 3.345398 0.418997 -0.214949

N -3.331218 -0.414076 -0.263993

N -0.413628 3.343355 -0.119159

N 0.420658 -3.340956 -0.111149

Co -0.004245 0.000096 -0.083799

C -0.150543 -0.016839 1.798138

O -1.211391 -0.045730 2.381105

O 0.994409 0.004872 2.517727

H 1.765244 -0.009867 1.930790

**3**

-1 2

N -1.702101 -0.929604 -0.232766

C -1.887133 -2.300999 -0.160726

C -2.954550 -0.332964 -0.227347

C -3.317560 -2.600048 -0.147926

C -3.986071 -1.367148 -0.194598

N 0.924516 -1.704988 -0.197771

C 0.336732 -2.953881 -0.161024

C 2.298101 -1.889275 -0.229645

C 2.597264 -3.291044 -0.231855

C 1.351413 -3.967649 -0.185270

N 1.701635 0.930985 -0.183740

C 2.958677 0.338948 -0.208086

C 1.886063 2.305343 -0.136063

C 3.986642 1.375010 -0.188874

C 3.315070 2.606191 -0.136659

N -0.924113 1.710902 -0.225575

C -2.292994 1.890858 -0.242905

C -0.337271 2.958150 -0.171010

C -1.353248 3.973101 -0.184380

C -2.596962 3.294064 -0.232275

C 3.738746 -5.395625 -0.247303

H 4.655215 -5.977550 -0.269085

C 2.493612 -6.071987 -0.203085

C 3.804291 -4.009978 -0.263128

H 4.758782 -3.494612 -0.297595

C 1.296408 -5.371446 -0.173411

H 0.344159 -5.890937 -0.139485

H 2.482318 -7.157677 -0.192122

C -5.416131 -3.739934 -0.111237

H -5.994874 -4.657761 -0.078795

C -6.086402 -2.504300 -0.157870

C -4.021534 -3.803205 -0.106555

H -3.503089 -4.755533 -0.071260

C -5.379243 -1.301393 -0.200833

H -5.895951 -0.348207 -0.237374

H -7.171871 -2.488337 -0.160677

C -2.500124 6.074630 -0.182327

H -2.490804 7.160250 -0.163226

C -3.743094 5.396288 -0.229521

C -1.300698 5.375983 -0.160210

H -0.349595 5.897504 -0.124082

C -3.805020 4.009767 -0.255818

H -4.758451 3.492517 -0.292313

H -4.661114 5.975966 -0.245405

C 6.084556 2.516746 -0.175739

H 7.169963 2.503547 -0.189141

C 5.411468 3.751072 -0.124367

C 5.380136 1.312376 -0.209932

H 5.898589 0.360318 -0.250660

C 4.017245 3.811259 -0.106058

H 3.496934 4.762418 -0.067587

H 5.988412 4.670247 -0.098837

N 3.247840 -0.937333 -0.234618

N -3.242784 0.942380 -0.245368

N 0.973820 3.243089 -0.119529

N -0.975736 -3.238937 -0.123301

Co -0.006674 0.003400 -0.085568

C -0.105208 0.019692 1.789133

O -1.068979 0.400778 2.421681

O 0.980570 -0.422825 2.479376

H 1.658187 -0.734616 1.859623

**TS3**

-1 2

N -0.07885800 -1.90313000 -0.90132300

C 1.15021400 -2.48076500 -1.11136800

C -0.99553400 -2.91020900 -0.72161500

C 1.02181100 -3.93205400 -1.09175100

C -0.33649500 -4.20390500 -0.84314400

N 1.44269300 0.38143600 -1.10372200

C 2.43218300 -0.55363900 -1.28728100

C 2.01433100 1.62817400 -1.19040500

C 3.44527800 1.49778400 -1.42919100

C 3.71044800 0.11665400 -1.48841400

N -0.80446100 1.91403700 -0.68836400

C 0.11458600 2.92276600 -0.86199900

C -2.03070600 2.49190200 -0.46010800

C -0.54792300 4.21515600 -0.75082300

C -1.90432200 3.94262500 -0.49343100

N -2.32451000 -0.37090100 -0.45539700

C -2.89153400 -1.61848000 -0.35105300

C -3.30944400 0.56372100 -0.24422700

C -4.58084600 -0.10760400 -0.00995100

C -4.31633300 -1.48835800 -0.07732900

C 5.76290300 1.95620300 -1.76954800

H 6.58261500 2.65883700 -1.88125400

C 6.02910200 0.57524100 -1.82311200

C 4.46771200 2.43585000 -1.57192600

H 4.26383200 3.50019700 -1.52592200

C 5.00627400 -0.36351200 -1.68389400

H 5.21087900 -1.42795400 -1.72401000

H 7.04931800 0.23709900 -1.97429100

C 1.47822400 -6.27392600 -1.16176500

H 2.17138300 -7.10059800 -1.28131800

C 0.11940400 -6.54589600 -0.91446500

C 1.94751600 -4.96352800 -1.25381600

H 2.99489700 -4.75379700 -1.44249100

C -0.80600200 -5.51469000 -0.75232100

H -1.85270400 -5.72451800 -0.55999100

H -0.21130300 -7.57755500 -0.84787700

C -6.88171900 -0.56981000 0.42627200

H -7.89421600 -0.23310400 0.62533500

C -6.61706300 -1.95073200 0.35915700

C -5.86749000 0.37002100 0.24185400

H -6.07176700 1.43408100 0.29343400

C -5.33119400 -2.42831300 0.10558500

H -5.12719900 -3.49241500 0.05375800

H -7.42999700 -2.65440400 0.50787700

C -1.00554100 6.55687200 -0.68571200

H -0.67597500 7.58862700 -0.75626400

C -2.36259400 6.28434700 -0.42924600

C -0.08033600 5.52603300 -0.85037700

H 0.96531400 5.73578500 -1.04834800

C -2.83017100 4.97384800 -0.33059100

H -3.87597800 4.76397500 -0.13353300

H -3.05554100 7.11074800 -0.30662500

N 1.41222900 2.80359400 -1.09475000

N -2.28967800 -2.79210700 -0.46494800

N -3.18575500 1.88200800 -0.24125900

N 2.31089700 -1.87155400 -1.29669200

Co -0.41867500 0.00135200 -0.66208000

C -0.26382400 -0.00652100 1.46208600

O -0.66171400 0.31651500 2.49060200

O 1.30716100 -1.56760800 2.05403300

H 1.36472500 -2.37367300 1.52370700

O 2.09465600 2.20475400 2.09366300

H 1.22337700 2.10592400 1.68880400

H 2.56744700 1.35328000 1.88207000

O 3.30946600 -0.14542100 1.84018400

H 3.84284100 -0.38871100 1.07184200

H 2.45853000 -0.81122500 1.86877400

O 1.84057500 1.32580000 4.73768800

H 1.81930000 1.66228900 3.81387600

H 2.67169700 0.80555100 4.75291300

O 3.85213300 -0.64644600 4.47545700

H 3.81839900 -0.48410600 3.50320900

H 3.02060600 -1.14846600 4.62514800

O 1.17443000 -1.47277300 4.65358900

H 1.13026200 -0.49789000 4.71767100

H 1.18644200 -1.61825600 3.65659600

**3et**

-2 3

N 0.003030 -1.940969 -0.159214

C 1.127209 -2.759675 -0.099634

C -1.120879 -2.761576 -0.151510

C 0.710558 -4.138104 -0.050341

C -0.705745 -4.139838 -0.082511

N 1.954006 0.001085 -0.147125

C 2.777030 -1.126028 -0.193290

C 2.775653 1.129190 -0.193324

C 4.149303 0.711676 -0.288819

C 4.150170 -0.706825 -0.288778

N 0.000637 1.940808 -0.160554

C 1.123867 2.760826 -0.099921

C -1.124222 2.760129 -0.153197

C 0.705577 4.138748 -0.050394

C -0.710691 4.138856 -0.083412

N -1.941868 -0.001228 -0.338465

C -2.760622 -1.123177 -0.304157

C -2.761997 1.119748 -0.305502

C -4.142855 0.705740 -0.332692

C -4.142003 -0.710816 -0.331734

C 6.542555 0.712186 -0.454327

H 7.487730 1.243072 -0.520457

C 6.543422 -0.704385 -0.454280

C 5.354518 1.427489 -0.373218

H 5.355481 2.513313 -0.374728

C 5.356268 -1.421144 -0.373081

H 5.358568 -2.506967 -0.374551

H 7.489241 -1.234130 -0.520374

C 0.710130 -6.535203 0.032926

H 1.240822 -7.481731 0.078594

C -0.705158 -6.536720 0.000861

C 1.426196 -5.344215 0.005518

H 2.511780 -5.345113 0.027351

C -1.421126 -5.346740 -0.059195

H -2.506607 -5.349255 -0.086303

H -1.235645 -7.484159 0.023374

C -6.541190 0.703611 -0.347444

H -7.489038 1.233884 -0.352483

C -6.540335 -0.711669 -0.346454

C -5.349956 1.420691 -0.339942

H -5.352451 2.506539 -0.337169

C -5.348215 -1.427271 -0.338014

H -5.349351 -2.513118 -0.333757

H -7.487546 -1.243082 -0.350778

C 0.702302 6.535790 0.034244

H 1.231861 7.482908 0.080833

C -0.712968 6.535681 0.001296

C 1.419776 5.345654 0.006523

H 2.505342 5.347775 0.029131

C -1.427517 5.344899 -0.059835

H -2.512982 5.346159 -0.087599

H -1.244553 7.482498 0.024042

N 2.404231 2.403134 -0.132950

N -2.396385 -2.398535 -0.220496

N -2.399284 2.395634 -0.222546

N 2.407164 -2.400429 -0.132898

Co -0.017034 -0.000048 -0.068465

C -0.144893 0.000784 1.801401

O -1.169844 0.001279 2.457943

O 1.046356 0.000688 2.464915

H 1.739493 0.000617 1.773739

**3pt**

0 4

N 1.914537 0.204676 -0.184262

C 2.612977 1.397115 -0.140119

C 2.832856 -0.828842 -0.173048

C 4.056464 1.119386 -0.129742

C 4.194217 -0.274670 -0.151671

N -0.214803 1.951534 -0.095077

C 0.812847 2.864836 -0.124117

C -1.400205 2.647888 -0.102225

C -1.132811 4.067897 -0.147220

C 0.274156 4.205803 -0.159995

N -1.959260 -0.176244 -0.073057

C -2.877333 0.856742 -0.067030

C -2.657524 -1.367553 -0.074869

C -4.239381 0.302908 -0.064164

C -4.101590 -1.091077 -0.068017

N 0.167726 -1.922980 -0.145383

C 1.354146 -2.619494 -0.183378

C -0.860152 -2.836605 -0.135547

C -0.321554 -4.177066 -0.186156

C 1.085224 -4.038831 -0.216793

C -1.364008 6.446014 -0.220570

H -1.984462 7.336236 -0.244649

C 0.037880 6.583451 -0.232884

C -1.966453 5.188187 -0.178254

H -3.046227 5.084345 -0.169630

C 0.873758 5.466639 -0.203598

H 1.953124 5.574664 -0.213792

H 0.473008 7.577228 -0.266121

C 6.430414 1.353427 -0.094256

H 7.318620 1.976510 -0.070623

C 6.568174 -0.040319 -0.116062

C 5.164813 1.955930 -0.102017

H 5.056992 3.034938 -0.085100

C 5.445047 -0.878475 -0.146341

H 5.551016 -1.957701 -0.163127

H 7.561281 -0.477793 -0.108841

C -0.086999 -6.554977 -0.253325

H -0.522558 -7.549009 -0.267552

C 1.314795 -6.417288 -0.283324

C -0.921871 -5.438261 -0.205305

H -2.001017 -5.546363 -0.182148

C 1.917935 -5.159466 -0.266406

H 2.997463 -5.055460 -0.289545

H 1.934426 -7.307663 -0.319600

C -6.613486 0.067857 -0.072503

H -7.606718 0.505119 -0.074457

C -6.475625 -1.325962 -0.076232

C -5.490198 0.906521 -0.067926

H -5.596302 1.985873 -0.067853

C -5.209720 -1.928142 -0.075745

H -5.101788 -3.007262 -0.081565

H -7.363859 -1.949449 -0.080905

N -2.650673 2.145993 -0.072209

N 2.604675 -2.117632 -0.175571

N -2.183701 -2.588256 -0.090410

N 2.137654 2.616035 -0.113744

Co -0.020015 0.013558 0.018417

C 0.228000 -0.127211 2.074858

O 0.661947 -0.417544 3.087518

**4**

-1 3

N 1.871536 0.442759 -0.191445

C 2.418683 1.713394 -0.135464

C 2.909942 -0.473948 -0.193631

C 3.878156 1.611355 -0.132697

C 4.185764 0.241531 -0.172294

N -0.461773 1.922972 -0.092917

C 0.449962 2.956016 -0.106231

C -1.725734 2.469148 -0.097692

C -1.631999 3.903232 -0.125395

C -0.246245 4.213246 -0.128424

N -1.935888 -0.410624 -0.072264

C -2.973930 0.504849 -0.071531

C -2.482778 -1.680645 -0.071041

C -4.250590 -0.210447 -0.068274

C -3.942860 -1.580505 -0.065055

N 0.394827 -1.892656 -0.166232

C 1.660302 -2.438473 -0.199914

C -0.516597 -2.925753 -0.133789

C 0.180076 -4.182069 -0.169336

C 1.565389 -3.871713 -0.212176

C -2.153831 6.241371 -0.178980

H -2.876498 7.051692 -0.199495

C -0.770990 6.550818 -0.182332

C -2.594794 4.925273 -0.151295

H -3.654674 4.690392 -0.150672

C 0.188923 5.548250 -0.158443

H 1.247846 5.787424 -0.162897

H -0.462604 7.591780 -0.204784

C 6.210038 2.135347 -0.104360

H 7.017282 2.860819 -0.076729

C 6.518129 0.763577 -0.144319

C 4.885260 2.575885 -0.099187

H 4.646644 3.633906 -0.068290

C 5.508811 -0.200319 -0.179837

H 5.745558 -1.258786 -0.210260

H 7.558136 0.452186 -0.147194

C 0.703950 -6.520110 -0.207871

H 0.395855 -7.561378 -0.206378

C 2.086498 -6.210365 -0.250472

C -0.255100 -5.517663 -0.168106

H -1.313556 -5.756717 -0.135859

C 2.527544 -4.894440 -0.254170

H 3.586866 -4.659373 -0.286887

H 2.808450 -7.021024 -0.280413

C -6.582807 -0.733503 -0.071008

H -7.622895 -0.422328 -0.073316

C -6.274502 -2.105660 -0.067573

C -5.573526 0.231305 -0.072727

H -5.810518 1.290150 -0.077571

C -4.949433 -2.545884 -0.066346

H -4.710684 -3.604331 -0.065981

H -7.081593 -2.831843 -0.066817

N -2.901825 1.813550 -0.077380

N 2.835944 -1.782117 -0.203967

N -1.858771 -2.833836 -0.084500

N 1.793366 2.864284 -0.098466

Co -0.029036 0.014179 0.005057

C 0.327478 -0.142376 2.034916

O 0.934164 -0.450009 2.952192

**TS4**

0 2

N -1.688645 0.511392 -0.813064

C -2.695823 -0.414370 -0.930614

C -2.278313 1.751615 -0.728633

C -3.997307 0.250685 -0.923100

C -3.733105 1.622278 -0.788872

N -0.168290 -1.759788 -0.816637

C -1.405966 -2.337363 -0.958584

C 0.755718 -2.777909 -0.759105

C 0.087765 -4.071725 -0.884639

C -1.281118 -3.792598 -1.014746

N 2.087491 -0.251663 -0.561614

C 2.667759 -1.496925 -0.533156

C 3.093549 0.674252 -0.419136

C 4.116707 -1.374280 -0.388164

C 4.386635 0.000924 -0.317427

N 0.566952 2.020189 -0.571281

C -0.361149 3.033756 -0.568780

C 1.807947 2.599032 -0.448316

C 1.681757 4.054307 -0.397674

C 0.308007 4.329445 -0.474338

C -0.376395 -6.405124 -1.022190

H -0.041642 -7.437112 -1.025876

C -1.743778 -6.126157 -1.151368

C 0.565347 -5.376456 -0.886583

H 1.622938 -5.592252 -0.784191

C -2.219552 -4.808170 -1.149696

H -3.277498 -4.591860 -1.247443

H -2.446574 -6.946481 -1.253142

C -6.337079 0.702013 -0.926235

H -7.365791 0.361008 -0.976752

C -6.073591 2.071660 -0.788349

C -5.297148 -0.234448 -0.996200

H -5.500584 -1.294534 -1.098020

C -4.760278 2.555479 -0.718114

H -4.556008 3.614742 -0.609639

H -6.902443 2.769651 -0.733617

C 2.141100 6.389408 -0.271112

H 2.844389 7.211784 -0.191616

C 0.768907 6.664130 -0.347118

C 2.620957 5.073086 -0.295143

H 3.682462 4.860469 -0.235277

C -0.173715 5.632495 -0.450329

H -1.235427 5.844553 -0.508368

H 0.430914 7.694845 -0.325220

C 6.444279 -1.841100 -0.178470

H 7.265596 -2.547767 -0.121022

C 6.713965 -0.467570 -0.108686

C 5.134372 -2.317914 -0.320890

H 4.925422 -3.380439 -0.375260

C 5.683598 0.479520 -0.178209

H 5.892122 1.542160 -0.124448

H 7.739880 -0.132035 0.001293

N 2.065986 -2.672084 -0.621562

N -1.678530 2.924760 -0.626790

N 2.979629 1.990898 -0.371365

N -2.580475 -1.730014 -1.024230

Co 0.197343 0.127975 -0.582379

C 0.063568 0.034853 1.338192

O 0.865619 0.569795 2.043780

H -0.977554 -0.641797 1.638839

O -2.047779 -1.293558 2.098944

H -0.446728 -0.775019 4.662514

H -2.606470 -1.641845 1.390723

O 0.530712 -0.878111 4.560898

H 0.746308 -0.240679 3.860344

O -2.221978 -0.654824 4.666073

H -2.373673 0.277284 4.439535

H -2.278035 -1.072690 3.768354

O 0.155995 -2.882337 2.600100

H 0.424114 -2.269571 3.317760

H -0.716012 -2.502027 2.354663

O -2.661360 1.357739 2.448606

H -2.560091 0.397647 2.239841

H -3.522093 1.590206 2.073564

**TS5**

0 4

N -1.70355300 0.90321800 -0.15473500

C -2.93540700 0.32509000 -0.13057100

C -1.87591900 2.29120700 -0.16025800

C -3.96875900 1.36213800 -0.13928400

C -3.29588800 2.59813200 -0.15698600

N -0.93699200 -1.72860400 -0.10573700

C -2.28551800 -1.90106700 -0.11604500

C -0.33951500 -2.99229900 -0.10727900

C -1.37305700 -4.01295300 -0.12796700

C -2.60342500 -3.32948400 -0.13504700

N 1.72077600 -0.95573300 -0.09972100

C 1.89359900 -2.34299400 -0.09183700

C 2.95168900 -0.37857600 -0.09870000

C 3.31374600 -2.65080000 -0.08959100

C 3.98622800 -1.41465400 -0.09467500

N 0.95564000 1.67641100 -0.15310600

C 0.35768900 2.94039200 -0.17172500

C 2.30540800 1.84832200 -0.13953500

C 2.62236400 3.27673100 -0.17225000

C 1.39188900 3.96001800 -0.19054700

C -2.54250500 -6.09624600 -0.16582000

H -2.53964600 -7.18161000 -0.17754400

C -3.76914400 -5.41261500 -0.17284900

C -1.32648500 -5.40633600 -0.14430500

H -0.38132800 -5.93839100 -0.13948000

C -3.81409800 -4.01400800 -0.15810100

H -4.75997500 -3.48322600 -0.16442000

H -4.69474400 -5.97878500 -0.19028900

C -6.06894300 2.49751900 -0.14265100

H -7.15395700 2.48006900 -0.13649600

C -5.39913500 3.73158600 -0.16119100

C -5.35791900 1.29219900 -0.13247500

H -5.87287600 0.33773800 -0.11904400

C -4.00263100 3.79989100 -0.16974000

H -3.48880100 4.75498100 -0.18468600

H -5.97805700 4.64967800 -0.16892400

C 3.78853300 5.35947100 -0.21714900

H 4.71424500 5.92561100 -0.22765400

C 2.56181300 6.04291300 -0.23577800

C 3.83325200 3.96123500 -0.18550300

H 4.77906000 3.43047800 -0.17175100

C 1.34567200 5.35322500 -0.22382500

H 0.40052800 5.88509400 -0.23872400

H 2.55915700 7.12806700 -0.26000900

C 5.41709500 -3.78399100 -0.08817000

H 5.99613600 -4.70203600 -0.08506400

C 6.08658300 -2.54974900 -0.09286400

C 4.02042600 -3.85248000 -0.08745900

H 3.50670400 -4.80776100 -0.08459000

C 5.37531300 -1.34438600 -0.09732200

H 5.89026700 -0.38983200 -0.10221100

H 7.17161900 -2.53198000 -0.09363800

N 0.95273600 -3.27056300 -0.09163700

N -0.93463500 3.21822300 -0.16693500

N 3.26134200 0.91925900 -0.10279200

N -3.24351200 -0.97125300 -0.10726300

Co 0.00781700 -0.02511900 0.00152300

C -0.08335600 0.24446100 2.07149300

O -0.26494800 0.76901200 3.06661200

**TS6**

-1 3

N 1.58030900 1.06240700 -0.19242700

C 1.66599700 2.44397600 -0.15480500

C 2.86903200 0.55427900 -0.21162200

C 3.07286400 2.84378900 -0.16631000

C 3.82695100 1.65918000 -0.20326700

N -1.11195700 1.66409400 -0.13241000

C -0.60555700 2.94687500 -0.11076900

C -2.48861600 1.75092200 -0.10694800

C -2.88516700 3.13160500 -0.07349000

C -1.68677500 3.89288100 -0.07423100

N -1.70914100 -1.02741300 -0.13444500

C -2.99744600 -0.51910200 -0.10936900

C -1.79495200 -2.40881500 -0.11802200

C -3.95557300 -1.62385600 -0.08228900

C -3.20149200 -2.80885700 -0.08591300

N 0.98208700 -1.62878500 -0.19802600

C 2.35965400 -1.71565500 -0.21706900

C 0.47633400 -2.91140700 -0.15892400

C 1.55824100 -3.85726500 -0.16759500

C 2.75624400 -3.09603100 -0.20518900

C -4.16835200 5.15395700 -0.00085500

H -5.12314300 5.67042600 0.02925500

C -2.97258000 5.91369900 -0.00188300

C -4.13728800 3.76621300 -0.03685500

H -5.05487300 3.18615500 -0.03483800

C -1.72945700 5.29601000 -0.03846900

H -0.81485500 5.88077400 -0.03827000

H -3.03434500 6.99749600 0.02739000

C 5.08844000 4.12856800 -0.17295600

H 5.60121600 5.08542500 -0.16208100

C 5.84346000 2.94267900 -0.20979100

C 3.69274500 4.09303100 -0.15035600

H 3.10920600 5.00737700 -0.12232300

C 5.22112800 1.69279600 -0.22522800

H 5.80337700 0.77764400 -0.25404300

H 6.92734300 3.00253100 -0.22704900

C 2.84502200 -5.87875400 -0.16620300

H 2.90745200 -6.96281100 -0.15246300

C 4.04044900 -5.11896700 -0.20199100

C 1.60176800 -5.26080300 -0.14858900

H 0.68760000 -5.84557400 -0.12114800

C 4.00875000 -3.73110500 -0.22188400

H 4.92587400 -3.15102700 -0.25007900

H 4.99538500 -5.63587300 -0.21497900

C -5.97151700 -2.90681300 -0.02149300

H -7.05524500 -2.96621400 0.00497900

C -5.21641200 -4.09314500 -0.02591600

C -5.34951700 -1.65691500 -0.04956500

H -5.93195600 -0.74143700 -0.04505700

C -3.82085600 -4.05808600 -0.05863700

H -3.23717400 -4.97277300 -0.06124000

H -5.72889900 -5.04994700 -0.00301200

N -3.37263100 0.73668000 -0.09961100

N 3.24348700 -0.70152700 -0.22920300

N -0.81689600 -3.28173900 -0.12452300

N 0.68832300 3.31629000 -0.11721100

Co -0.06344100 0.01710000 -0.14238000

C 0.76104200 -0.22491700 2.43502600

O 1.76393500 -0.47046400 2.91411900

**TS7**

-1 1

N -1.664277 0.599820 -0.815641

C -2.713789 -0.279328 -0.934906

C -2.191600 1.869166 -0.740383

C -3.975874 0.447090 -0.937247

C -3.645089 1.808536 -0.810140

N -0.247486 -1.750668 -0.822707

C -1.513915 -2.266790 -0.961284

C 0.628815 -2.810934 -0.766304

C -0.099943 -4.065989 -0.887706

C -1.457539 -3.721129 -1.013692

N 2.084453 -0.347124 -0.554964

C 2.603700 -1.620560 -0.537573

C 3.133186 0.532138 -0.413744

C 4.052231 -1.565085 -0.398744

C 4.387844 -0.201462 -0.321150

N 0.669451 2.003799 -0.565951

C -0.212003 3.059684 -0.561281

C 1.937955 2.520209 -0.435260

C 1.878901 3.974086 -0.373453

C 0.516935 4.315627 -0.453585

C -0.675140 -6.378869 -1.018346

H -0.391857 -7.426676 -1.021052

C -2.034115 -6.033545 -1.143460

C 0.310066 -5.399677 -0.889578

H 1.357008 -5.665768 -0.790883

C -2.443434 -4.699948 -1.143419

H -3.490712 -4.433552 -1.238155

H -2.774629 -6.821232 -1.240601

C -6.295124 1.011104 -0.956246

H -7.339869 0.721673 -1.009889

C -5.964706 2.373147 -0.824759

C -5.305135 0.030033 -1.014683

H -5.559692 -1.020001 -1.110804

C -4.635384 2.790211 -0.750734

H -4.380854 3.839564 -0.647415

H -6.760523 3.109848 -0.778541

C 2.450387 6.287161 -0.226716

H 3.190532 7.076186 -0.137856

C 1.087134 6.628901 -0.306811

C 2.864396 4.955383 -0.259322

H 3.914958 4.692291 -0.197277

C 0.102314 5.647617 -0.421544

H -0.948265 5.910635 -0.483134

H 0.800057 7.675307 -0.278389

C 6.359267 -2.145090 -0.201162

H 7.148040 -2.889118 -0.150855

C 6.695305 -0.780250 -0.124091

C 5.033315 -2.555635 -0.340328

H 4.774104 -3.607293 -0.400003

C 5.714211 0.209685 -0.183991

H 5.973199 1.261405 -0.124793

H 7.737295 -0.495903 -0.016246

N 1.943229 -2.763642 -0.630746

N -1.532837 3.009935 -0.629808

N 3.077534 1.852623 -0.360590

N -2.657099 -1.600147 -1.020669

Co 0.208314 0.125251 -0.571902

C 0.065439 0.037725 1.340385

O 0.877119 0.553828 2.055343

H -1.026615 -0.638916 1.649572

O -2.073946 -1.253926 2.063634

H -0.529732 -0.743749 4.654461

H -2.593162 -1.568400 1.309894

O 0.447567 -0.848432 4.562392

H 0.669521 -0.233244 3.842715

O -2.315604 -0.642420 4.645017

H -2.480316 0.288817 4.424207

H -2.351342 -1.053972 3.745719

O 0.078465 -2.899271 2.644582

H 0.341896 -2.271906 3.351040

H -0.775308 -2.505494 2.363323

O -2.721111 1.397138 2.452668

H -2.598427 0.445436 2.224825

H -3.532176 1.645470 1.987460

**4et**

-2 2

N -1.905128 -0.311770 -0.200414

C -2.544811 -1.547063 -0.126435

C -2.878324 0.684050 -0.165707

C -3.997082 -1.332183 -0.089165

C -4.204442 0.055963 -0.114005

N 0.323770 -1.962136 -0.088975

C -0.663549 -2.929073 -0.132289

C 1.549743 -2.598928 -0.103313

C 1.354084 -4.003301 -0.170001

C -0.065756 -4.214906 -0.187264

N 1.970181 0.267189 -0.069507

C 2.942476 -0.727192 -0.039336

C 2.609545 1.501268 -0.060340

C 4.269849 -0.099173 -0.007454

C 4.062758 1.288871 -0.019742

N -0.255847 1.918704 -0.161437

C -1.483577 2.555494 -0.209874

C 0.732359 2.885047 -0.159553

C 0.135101 4.170276 -0.232971

C -1.284782 3.958780 -0.263632

C 1.704833 -6.381857 -0.279840

H 2.367685 -7.242035 -0.317020

C 0.294573 -6.591988 -0.296824

C 2.235848 -5.105716 -0.217978

H 3.311615 -4.952317 -0.206790

C -0.586612 -5.526302 -0.252453

H -1.660366 -5.693017 -0.267027

H -0.087635 -7.607934 -0.346218

C -6.363583 -1.684492 -0.008900

H -7.219791 -2.351104 0.033062

C -6.570998 -0.295307 -0.033551

C -5.071895 -2.218467 -0.037319

H -4.910261 -3.291633 -0.018062

C -5.491346 0.591198 -0.087457

H -5.650235 1.664758 -0.106345

H -7.584753 0.093207 -0.010035

C -0.220748 6.547944 -0.339746

H 0.162800 7.564077 -0.370545

C -1.631183 6.338076 -0.368050

C 0.658200 5.481812 -0.273951

H 1.731904 5.647883 -0.252833

C -2.164491 5.062340 -0.332082

H -3.240102 4.909421 -0.354818

H -2.292221 7.198959 -0.418760

C 6.636857 0.253659 0.041182

H 7.650858 -0.134201 0.065545

C 6.429618 1.643059 0.028712

C 5.556858 -0.633843 0.021466

H 5.715746 -1.707560 0.028642

C 5.137652 2.176217 -0.003854

H 4.976150 3.249523 -0.015949

H 7.286189 2.310368 0.043615

N 2.787526 -2.018612 -0.049896

N -2.720872 1.974654 -0.177627

N 2.084835 2.691544 -0.093755

N -2.017246 -2.734902 -0.101962

Co 0.026586 -0.019720 0.043825

C -0.340964 0.202652 2.007480

O -0.952896 0.632487 2.877592

**4pt**

0 1

N -1.427888 1.328267 -0.144584

C -2.781396 1.074281 -0.095891

C -1.260748 2.695731 -0.147147

C -3.519526 2.327154 -0.095589

C -2.557997 3.352348 -0.132048

N -1.344366 -1.412735 -0.138871

C -2.710563 -1.241631 -0.086476

C -1.094501 -2.767385 -0.135571

C -2.348982 -3.501813 -0.108731

C -3.371052 -2.536872 -0.074547

N 1.385242 -1.328592 -0.167932

C 1.217927 -2.696029 -0.159940

C 2.738333 -1.074940 -0.137067

C 2.515458 -3.353213 -0.157258

C 3.477092 -2.328042 -0.138000

N 1.301847 1.410510 -0.170368

C 1.051870 2.765193 -0.157273

C 2.667618 1.239670 -0.131245

C 3.328736 2.535111 -0.117185

C 2.306654 3.500052 -0.137622

C -3.984979 -5.237611 -0.074588

H -4.249899 -6.290095 -0.073483

C -5.008753 -4.271060 -0.039951

C -2.641480 -4.866686 -0.110169

H -1.852956 -5.610875 -0.137588

C -4.716284 -2.907854 -0.040804

H -5.504545 -2.163352 -0.014516

H -6.043734 -4.596583 -0.012260

C -5.259840 3.958677 -0.078508

H -6.312832 4.220753 -0.057070

C -4.296696 4.985541 -0.115553

C -4.884901 2.615840 -0.069106

H -5.626428 1.824799 -0.041017

C -2.932980 4.696772 -0.143433

H -2.190868 5.487224 -0.172120

H -4.625373 6.019864 -0.122187

C 4.966655 4.269113 -0.086254

H 6.001854 4.594428 -0.065074

C 3.942769 5.235709 -0.106619

C 4.674049 2.905771 -0.092758

H 5.462465 2.161132 -0.077030

C 2.598901 4.864812 -0.133353

H 1.810143 5.609103 -0.149284

H 4.207621 6.288200 -0.101112

C 4.254182 -4.986359 -0.152011

H 4.582704 -6.020749 -0.156175

C 5.217522 -3.959457 -0.132813

C 2.890061 -4.697579 -0.165233

H 2.147668 -5.488169 -0.179781

C 4.842517 -2.616409 -0.126915

H 5.584267 -1.825207 -0.112326

H 6.270732 -4.221333 -0.122551

N 0.081662 -3.370137 -0.147850

N -0.123741 3.368887 -0.152720

N 3.338967 0.101557 -0.114174

N -3.380927 -0.102786 -0.060126

Co -0.017479 -0.000293 -0.062204

C -0.045099 0.003505 1.796595

O 0.927950 0.021542 2.505066

H -1.075460 -0.012439 2.207308

**TS8**

-2 2

N -1.666249 0.586158 -0.839081

C -2.709614 -0.308366 -0.945517

C -2.207886 1.859832 -0.750418

C -3.982975 0.412744 -0.929233

C -3.666764 1.776023 -0.802255

N -0.220506 -1.760821 -0.832669

C -1.482468 -2.288055 -0.984580

C 0.670206 -2.814803 -0.776182

C -0.036909 -4.058352 -0.912728

C -1.409988 -3.723045 -1.046263

N 2.107506 -0.327057 -0.549936

C 2.638161 -1.605834 -0.518338

C 3.147964 0.566996 -0.391446

C 4.089707 -1.529328 -0.357091

C 4.410838 -0.164152 -0.278437

N 0.664307 2.018532 -0.563376

C -0.231816 3.069704 -0.575699

C 1.928593 2.546926 -0.434878

C 1.855419 3.983119 -0.390041

C 0.478255 4.315250 -0.480075

C -0.593457 -6.384572 -1.066368

H -0.301052 -7.430439 -1.076037

C -1.963607 -6.049721 -1.197382

C 0.376210 -5.400238 -0.924496

H 1.425216 -5.660370 -0.821741

C -2.382346 -4.725219 -1.189332

H -3.433139 -4.470749 -1.288036

H -2.695144 -6.845187 -1.304553

C -6.312513 0.948758 -0.912823

H -7.354034 0.644928 -0.952479

C -5.996893 2.312779 -0.781141

C -5.307708 -0.018300 -0.988611

H -5.550216 -1.071646 -1.084026

C -4.669351 2.743209 -0.724628

H -4.425838 3.795505 -0.620630

H -6.799814 3.041082 -0.720502

C 2.408285 6.311828 -0.264653

H 3.141032 7.109024 -0.181261

C 1.034166 6.643346 -0.354165

C 2.829384 4.988012 -0.282186

H 3.883263 4.736349 -0.213705

C 0.062770 5.656389 -0.462408

H -0.989696 5.913842 -0.531048

H 0.739662 7.688550 -0.337635

C 6.402768 -2.086770 -0.123559

H 7.196898 -2.824450 -0.059791

C 6.724169 -0.720017 -0.045068

C 5.080616 -2.507967 -0.281557

H 4.832350 -3.562584 -0.342499

C 5.730563 0.258473 -0.122808

H 5.977637 1.313414 -0.062681

H 7.761345 -0.423370 0.077579

N 2.003391 -2.748219 -0.617879

N -1.572853 2.999593 -0.642277

N 3.092014 1.876885 -0.341776

N -2.652165 -1.619356 -1.031740

Co 0.217301 0.127614 -0.558656

C 0.060102 0.032393 1.337737

O 0.863190 0.547889 2.074729

H -1.068973 -0.676918 1.647983

O -2.087582 -1.281096 2.007235

H -0.620580 -0.713462 4.629489

H -2.558268 -1.579277 1.214078

O 0.358967 -0.804078 4.557138

H 0.584765 -0.213197 3.816068

O -2.416732 -0.660767 4.589550

H -2.598350 0.260380 4.337636

H -2.418015 -1.092701 3.701176

O 0.049127 -2.921236 2.701850

H 0.287766 -2.265768 3.391441

H -0.789909 -2.537267 2.370517

O -2.769845 1.362948 2.445953

H -2.630829 0.422982 2.184318

H -3.571637 1.620233 1.969311

**TS9**

-2 2

N 1.895442 -0.610306 -0.104610

C 3.036162 0.188905 -0.092094

C 2.302617 -1.940609 -0.108625

C 4.226737 -0.669846 -0.082703

C 3.770087 -1.996772 -0.091008

N 0.686616 1.876923 -0.114639

C 2.008274 2.282425 -0.085141

C -0.106375 3.010553 -0.105982

C 0.715947 4.167894 -0.072930

C 2.071917 3.700898 -0.060837

N -1.793711 0.662840 -0.239382

C -2.203580 1.994227 -0.167108

C -2.936840 -0.137529 -0.232966

C -3.670452 2.047616 -0.162573

C -4.126450 0.720973 -0.205996

N -0.587673 -1.826351 -0.208984

C 0.204333 -2.957590 -0.147739

C -1.910955 -2.231690 -0.227562

C -1.974722 -3.649535 -0.191348

C -0.618957 -4.114995 -0.141227

C 1.502193 6.441060 -0.018257

H 1.311289 7.510581 -0.000568

C 2.849877 5.977137 -0.006456

C 0.441525 5.552827 -0.051706

H -0.583914 5.912275 -0.060562

C 3.139918 4.624227 -0.027731

H 4.169497 4.276919 -0.018580

H 3.658212 6.702793 0.019479

C 6.490208 -1.448090 -0.057798

H 7.558104 -1.251362 -0.044795

C 6.033139 -2.776451 -0.066230

C 5.589953 -0.378596 -0.065986

H 5.941945 0.648151 -0.059204

C 4.665374 -3.065240 -0.083337

H 4.311063 -4.091187 -0.090157

H 6.753756 -3.588812 -0.059543

C -2.752273 -5.925877 -0.142254

H -3.560405 -6.652210 -0.139512

C -1.404790 -6.388397 -0.095268

C -3.042548 -4.573834 -0.190644

H -4.071934 -4.227725 -0.226224

C -0.344405 -5.499281 -0.094870

H 0.680873 -5.857341 -0.058254

H -1.214010 -7.457462 -0.059368

C -5.934524 2.824927 -0.127419

H -6.655681 3.636259 -0.096880

C -6.390815 1.496963 -0.171167

C -4.566919 3.114659 -0.122315

H -4.213317 4.140275 -0.087665

C -5.489723 0.428932 -0.210586

H -5.840891 -0.597592 -0.243154

H -7.458629 1.299434 -0.173945

N -1.470696 3.066416 -0.111302

N 1.569107 -3.015318 -0.119663

N -3.018826 -1.434782 -0.238565

N 3.118707 1.486886 -0.081350

Co 0.044289 0.025301 -0.119795

C -0.493745 -0.266365 2.239411

O -1.406957 -0.731430 2.746695

**5**

-1 2

N -0.395336 1.916134 -0.143424

C -1.649137 2.494134 -0.091501

C 0.535917 2.935422 -0.155289

C -1.524303 3.923845 -0.093334

C -0.134444 4.204710 -0.136697

N -1.919461 -0.374429 -0.140388

C -2.939209 0.563907 -0.081317

C -2.503014 -1.631457 -0.139069

C -3.957482 -1.494875 -0.111209

C -4.230722 -0.118737 -0.070073

N 0.360879 -1.892834 -0.167816

C -0.570480 -2.912108 -0.165397

C 1.614502 -2.471039 -0.139077

C 0.099782 -4.181890 -0.159066

C 1.489875 -3.901409 -0.138423

N 1.884269 0.396017 -0.190335

C 2.467576 1.653038 -0.171696

C 2.903895 -0.541911 -0.138100

C 4.195683 0.140463 -0.119628

C 3.922323 1.516791 -0.146215

C -6.300264 -1.962463 -0.081678

H -7.125209 -2.668185 -0.085595

C -6.574050 -0.583228 -0.040392

C -4.987488 -2.435578 -0.118134

H -4.775113 -3.499048 -0.150704

C -5.541799 0.356421 -0.035098

H -5.751931 1.420392 -0.004676

H -7.605798 -0.246723 -0.012874

C -1.996915 6.271708 -0.076323

H -2.702207 7.097073 -0.053285

C -0.608316 6.552250 -0.118968

C -2.466228 4.965575 -0.064145

H -3.530199 4.753185 -0.032221

C 0.330175 5.530127 -0.150069

H 1.393167 5.747147 -0.183017

H -0.277950 7.586579 -0.127501

C 6.538695 0.605223 -0.074443

H 7.570393 0.268658 -0.045552

C 6.264723 1.984720 -0.100755

C 5.506657 -0.334728 -0.084538

H 5.716945 -1.398902 -0.064002

C 4.951878 2.457852 -0.137162

H 4.739273 3.521600 -0.156649

H 7.089430 2.690680 -0.091973

C 0.573564 -6.529435 -0.149328

H 0.242868 -7.563701 -0.152494

C 1.962519 -6.249204 -0.129631

C -0.365278 -5.507068 -0.164498

H -1.428691 -5.723983 -0.179797

C 2.432008 -4.942851 -0.125146

H 3.496356 -4.730460 -0.110364

H 2.668048 -7.074599 -0.117961

N -1.909146 -2.798018 -0.154184

N 1.874496 2.820193 -0.165217

N 2.802982 -1.846408 -0.114761

N -2.837491 1.868291 -0.051027

Co -0.012920 0.009237 -0.054328

C -0.036530 0.036538 1.792899

O 0.757941 -0.505594 2.525941

H -0.887092 0.624544 2.199053

**TS10**

-1 2

N 1.882801 -1.096046 -0.517262

C 3.182555 -0.639452 -0.438807

C 1.912461 -2.468602 -0.465436

C 4.092994 -1.771016 -0.372557

C 3.290129 -2.926349 -0.382062

N 1.383214 1.601809 -0.510411

C 2.756833 1.637756 -0.401555

C 0.923592 2.896412 -0.489065

C 2.043309 3.813576 -0.349579

C 3.202850 3.017684 -0.300423

N -1.298357 1.101231 -0.785618

C -1.337722 2.478288 -0.770718

C -2.579283 0.648142 -0.990168

C -2.702313 2.934689 -0.996494

C -3.484222 1.778885 -1.147804

N -0.810106 -1.584614 -0.696207

C -0.360940 -2.883359 -0.601361

C -2.171670 -1.623039 -0.878511

C -2.628038 -3.006297 -0.898265

C -1.486639 -3.802024 -0.712600

C 3.381693 5.778771 -0.147118

H 3.476926 6.858369 -0.084298

C 4.542044 4.982537 -0.097565

C 2.119231 5.204951 -0.275451

H 1.220811 5.810959 -0.316352

C 4.467464 3.593969 -0.174744

H 5.356624 2.974211 -0.138278

H 5.510274 5.463277 0.002470

C 6.048718 -3.131071 -0.233981

H 7.127608 -3.236344 -0.174543

C 5.245285 -4.287322 -0.244925

C 5.483337 -1.859718 -0.296921

H 6.095253 -0.964442 -0.287238

C 3.857252 -4.199442 -0.319279

H 3.230792 -5.084563 -0.326533

H 5.719691 -5.262454 -0.193609

C -3.976100 -4.968025 -1.034052

H -4.942535 -5.447261 -1.157057

C -2.832783 -5.765783 -0.848907

C -3.887549 -3.576833 -1.062093

H -4.763057 -2.952964 -1.203051

C -1.572005 -5.192445 -0.688261

H -0.684757 -5.799989 -0.547845

H -2.937061 -6.846369 -0.832606

C -4.636309 4.288258 -1.335069

H -5.109901 5.262178 -1.412549

C -5.418869 3.129579 -1.487028

C -3.266688 4.205479 -1.088132

H -2.655650 5.093686 -0.970419

C -4.851997 1.859296 -1.393550

H -5.447820 0.960149 -1.502993

H -6.482841 3.230105 -1.678576

N -0.329034 3.314970 -0.612312

N 0.885034 -3.303391 -0.489814

N -2.994950 -0.604702 -1.051394

N 3.595808 0.614410 -0.387576

Co 0.279320 0.005322 -0.519556

C 0.749572 -0.305971 1.802102

O 0.421567 -1.348533 2.387135

H 1.820764 0.007525 1.920684

O -0.715751 1.689439 2.274951

H -0.354067 2.509350 1.915019

H -0.093881 0.705279 1.688078

O -3.256412 0.891766 1.966096

H -3.022349 -0.036458 1.757657

H -2.362819 1.315037 2.029553

O -0.422670 0.547891 4.657025

H -0.502977 1.128773 3.851882

H -0.059710 -0.264842 4.276667

O -2.341745 -1.762589 2.216091

H -2.598459 -1.453714 3.112905

H -1.368378 -1.659141 2.205068

O -3.070328 -0.282906 4.471611

H -3.310767 0.288440 3.707755

H -2.158653 0.022160 4.679390

**5et**

-2 1

N -0.199266 1.953072 -0.146298

C -1.390710 2.657532 -0.102278

C 0.835369 2.874643 -0.163770

C -1.122941 4.048892 -0.117464

C 0.303819 4.188124 -0.157707

N -1.953274 -0.177529 -0.144828

C -2.876373 0.865798 -0.075492

C -2.665196 -1.376685 -0.128543

C -4.102609 -1.085643 -0.089389

C -4.233953 0.309788 -0.051176

N 0.167372 -1.927476 -0.167096

C -0.866853 -2.848996 -0.168094

C 1.359325 -2.632465 -0.150059

C -0.335589 -4.162791 -0.177566

C 1.091392 -4.024309 -0.162876

N 1.920506 0.201005 -0.220825

C 2.632460 1.400117 -0.174329

C 2.844108 -0.841573 -0.142225

C 4.201401 -0.286049 -0.104601

C 4.069904 1.109512 -0.130647

C -6.484242 -1.312465 -0.041658

H -7.375775 -1.932351 -0.037287

C -6.615828 0.085952 -0.003582

C -5.223705 -1.914543 -0.085723

H -5.120963 -2.994572 -0.116109

C -5.489758 0.913860 -0.009162

H -5.590179 1.994172 0.018453

H -7.607291 0.527547 0.029757

C -1.352110 6.443476 -0.122895

H -1.969240 7.337640 -0.110515

C 0.067453 6.581826 -0.160932

C -1.948252 5.196114 -0.102366

H -3.029951 5.097495 -0.074164

C 0.892632 5.472482 -0.178967

H 1.973042 5.583741 -0.208582

H 0.500382 7.578238 -0.176068

C 6.582454 -0.061573 -0.026461

H 7.573665 -0.503056 0.015101

C 6.450614 1.337082 -0.051995

C 5.456984 -0.889922 -0.053127

H 5.557668 -1.970364 -0.032677

C 5.190249 1.938951 -0.104317

H 5.087136 3.019218 -0.122688

H 7.341655 1.957301 -0.030038

C -0.099846 -6.556547 -0.190746

H -0.533353 -7.552795 -0.200579

C 1.320043 -6.418787 -0.177845

C -0.925036 -5.446929 -0.191257

H -2.005821 -5.558012 -0.201566

C 1.916601 -5.171324 -0.165507

H 2.998645 -5.072810 -0.155987

H 1.937122 -7.313060 -0.177825

N -2.207077 -2.591074 -0.144091

N 2.175091 2.614910 -0.161449

N 2.623247 -2.120375 -0.114608

N -2.655301 2.144765 -0.050252

Co -0.010512 0.010050 -0.041481

C -0.033446 0.048252 1.793385

O 0.697415 -0.565098 2.545199

H -0.821479 0.719221 2.201533

**5pt**

0 2

N -1.880249 0.125341 -0.231222

C -2.767835 -0.928345 -0.235281

C -2.612640 1.291783 -0.214010

C -4.132353 -0.422769 -0.237835

C -4.034191 0.980995 -0.222201

N -0.077762 -1.944553 -0.170245

C -1.243800 -2.677179 -0.182963

C 0.974446 -2.830939 -0.128202

C 0.469699 -4.196345 -0.116147

C -0.933216 -4.099083 -0.150895

N 1.993683 -0.143109 -0.139457

C 2.725630 -1.308607 -0.109764

C 2.880494 0.909995 -0.131599

C 4.147586 -0.997970 -0.083998

C 4.245652 0.405192 -0.099165

N 0.193395 1.927976 -0.187112

C -0.860583 2.813463 -0.176875

C 1.357764 2.660481 -0.160592

C 1.046336 4.082575 -0.144422

C -0.357031 4.179315 -0.152406

C 0.304199 -6.576988 -0.076392

H 0.767041 -7.558313 -0.047319

C -1.099842 -6.479621 -0.111406

C 1.107683 -5.436976 -0.078389

H 2.189418 -5.512163 -0.051395

C -1.737291 -5.239671 -0.148997

H -2.819023 -5.164625 -0.175781

H -1.694484 -7.387608 -0.108829

C -6.513322 -0.256636 -0.238599

H -7.495173 -0.719220 -0.245107

C -6.415080 1.148028 -0.223142

C -5.373997 -1.060515 -0.245232

H -5.449880 -2.142473 -0.256028

C -5.174935 1.785404 -0.214160

H -5.099155 2.867320 -0.201280

H -7.322955 1.742804 -0.217953

C 1.210856 6.463254 -0.107032

H 1.804870 7.371483 -0.089270

C -0.193553 6.560149 -0.115722

C 1.849596 5.223378 -0.120831

H 2.931661 5.148704 -0.113690

C -0.996250 5.419733 -0.138368

H -2.078331 5.494586 -0.144797

H -0.657407 7.541367 -0.104424

C 6.527764 -1.164399 -0.028726

H 7.435303 -1.759072 -0.000787

C 6.625966 0.239874 -0.044110

C 5.287488 -1.802122 -0.048465

H 5.211708 -2.884062 -0.035940

C 5.486488 1.043435 -0.079395

H 5.562337 2.125406 -0.090567

H 7.607481 0.702937 -0.027652

N 2.267378 -2.549609 -0.101983

N -2.153463 2.532155 -0.187835

N 2.599347 2.202794 -0.141651

N -2.485397 -2.220988 -0.220892

Co 0.057386 -0.007667 -0.176111

C -1.502711 -0.092587 2.681078

O -0.425252 0.466582 2.795924

H -2.455878 0.459196 2.758689

H -1.577587 -1.179771 2.507964

**5pt-a**

0 2

N -1.839397 0.737237 -0.122992

C -3.003999 0.011381 -0.127100

C -2.171072 2.070350 -0.113403

C -4.148817 0.913240 -0.137569

C -3.620981 2.217829 -0.128641

N -0.759020 -1.794994 -0.133071

C -2.093990 -2.126700 -0.120083

C -0.031239 -2.961683 -0.127482

C -0.934175 -4.104171 -0.120937

C -2.238656 -3.576228 -0.117823

N 1.770635 -0.718663 -0.171038

C 2.105458 -2.052195 -0.168559

C 2.937277 0.011255 -0.191163

C 3.553910 -2.196022 -0.207087

C 4.080080 -0.890995 -0.224040

N 0.698823 1.813809 -0.119807

C -0.031132 2.978550 -0.102608

C 2.030208 2.149652 -0.143101

C 2.174539 3.599433 -0.137901

C 0.869016 4.124307 -0.111727

C -1.829094 -6.317099 -0.117532

H -1.692573 -7.393899 -0.116432

C -3.134053 -5.789045 -0.114299

C -0.711989 -5.481850 -0.122650

H 0.293579 -5.888570 -0.126645

C -3.356147 -4.411963 -0.115954

H -4.361717 -4.005266 -0.114959

H -3.980895 -6.468046 -0.110871

C -6.361516 1.808904 -0.161944

H -7.438245 1.672247 -0.174706

C -5.833689 3.113464 -0.152864

C -5.526022 0.691363 -0.155172

H -5.933145 -0.314059 -0.162988

C -4.456212 3.335227 -0.136820

H -4.049509 4.340823 -0.130681

H -6.512459 3.960487 -0.158853

C 3.064960 5.814290 -0.147957

H 3.910267 6.495102 -0.161019

C 1.759280 6.339299 -0.121818

C 3.289914 4.437443 -0.157988

H 4.296255 4.033159 -0.179962

C 0.644009 5.501322 -0.105030

H -0.362269 5.905942 -0.086650

H 1.620325 7.415779 -0.115275

C 5.766779 -3.089434 -0.263724

H 6.446525 -3.935546 -0.278723

C 6.293196 -1.783837 -0.280365

C 4.390477 -3.312860 -0.228029

H 3.984897 -4.318803 -0.215444

C 5.457095 -0.667550 -0.261650

H 5.862704 0.338387 -0.274921

H 7.369504 -1.646235 -0.308169

N 1.286266 -3.092292 -0.137164

N -1.349046 3.108409 -0.090494

N 3.070086 1.328666 -0.174902

N -3.133501 -1.307250 -0.114782

Co -0.028999 0.003784 0.015235

C 0.009588 -0.245011 2.006137

O 1.053636 -0.127200 2.793290

H -0.652109 -0.988824 2.483024

H 1.680797 0.524289 2.426678

**TS11**

-2 1

N -0.219576 1.984225 -0.663850

C -1.441183 2.607980 -0.636771

C 0.755516 2.962435 -0.579257

C -1.258905 4.044087 -0.535942

C 0.134892 4.268312 -0.502886

N -1.827410 -0.225153 -0.724132

C -2.802510 0.739136 -0.782209

C -2.452741 -1.458398 -0.780020

C -3.898634 -1.277113 -0.867502

C -4.120586 0.111034 -0.873904

N 0.401789 -1.839965 -0.670862

C -0.565116 -2.820042 -0.705189

C 1.628500 -2.474302 -0.668155

C 0.048513 -4.127388 -0.703266

C 1.445248 -3.910382 -0.685374

N 1.995987 0.369385 -0.661746

C 2.633863 1.600063 -0.591263

C 2.984500 -0.597915 -0.637732

C 4.302675 0.028575 -0.586747

C 4.079448 1.416016 -0.553234

C -6.255579 -1.653373 -1.001369

H -7.105249 -2.327604 -1.049766

C -6.477357 -0.264172 -1.010020

C -4.963164 -2.176724 -0.930898

H -4.792578 -3.248374 -0.923249

C -5.411023 0.635100 -0.946850

H -5.581235 1.706799 -0.950653

H -7.494400 0.111749 -1.065104

C -1.643020 6.401737 -0.390315

H -2.318554 7.250617 -0.345141

C -0.250337 6.625592 -0.351911

C -2.160951 5.112096 -0.484208

H -3.232117 4.939846 -0.513423

C 0.651255 5.565544 -0.408702

H 1.722110 5.740152 -0.380846

H 0.121424 7.643131 -0.277735

C 6.662762 0.401095 -0.496239

H 7.680470 0.023782 -0.472338

C 6.440432 1.789082 -0.464752

C 5.593879 -0.496648 -0.556899

H 5.763381 -1.568261 -0.579535

C 5.146177 2.313739 -0.492928

H 4.976235 3.385201 -0.467059

H 7.290561 2.462874 -0.416931

C 0.423215 -6.492264 -0.715535

H 0.046445 -7.510742 -0.725915

C 1.818321 -6.274588 -0.692613

C -0.473362 -5.427726 -0.723080

H -1.544944 -5.599209 -0.739285

C 2.342019 -4.984515 -0.678299

H 3.414193 -4.816027 -0.661816

H 2.489144 -7.128297 -0.686803

N -1.893499 -2.649643 -0.756966

N 2.072912 2.789736 -0.553058

N 2.831776 -1.905607 -0.649374

N -2.654020 2.051701 -0.748068

Co 0.105132 0.075406 -0.559267

C -0.195419 0.654443 1.592754

O 0.758947 1.243165 2.172671

H -1.145490 1.270046 1.589871

O -1.306056 -1.776130 2.200862

H -1.439021 -2.402365 1.474469

H -0.766645 -0.992577 1.801617

O 0.407067 -1.597444 4.339840

H 1.182271 -1.289201 3.816868

H -0.210856 -1.888188 3.636869

O -3.374425 -0.080216 2.677650

H -2.720915 -0.782670 2.445091

H -3.173910 0.623223 2.043679

O 2.418908 -0.747830 2.608497

H 3.192344 -0.407794 3.079043

H 1.874696 0.066701 2.401442

O -1.351863 0.625613 4.644915

H -0.707396 -0.105621 4.542360

H -2.062828 0.403754 4.013306

**6**

-1 3

N -1.781109 0.626147 -0.244986

C -2.920579 -0.154078 -0.233336

C -2.174259 1.949996 -0.229352

C -4.083290 0.689092 -0.223806

C -3.608279 2.027749 -0.220942

N -0.593014 -1.859665 -0.169707

C -1.920242 -2.253509 -0.187191

C 0.189957 -3.001947 -0.130008

C -0.671663 -4.183517 -0.127650

C -1.995106 -3.714360 -0.163494

N 1.894818 -0.673978 -0.126279

C 2.287967 -1.997349 -0.097229

C 3.032894 0.105423 -0.113098

C 3.722218 -2.075110 -0.064642

C 4.196565 -0.737110 -0.074528

N 0.709261 1.811792 -0.198063

C -0.075293 2.953337 -0.196494

C 2.035244 2.206039 -0.160306

C 2.109401 3.667095 -0.150976

C 0.785491 4.135764 -0.171596

C -1.470643 -6.436221 -0.102961

H -1.285872 -7.505811 -0.079734

C -2.795661 -5.966409 -0.139015

C -0.391885 -5.549550 -0.096548

H 0.630548 -5.911849 -0.068402

C -3.073744 -4.598317 -0.169526

H -4.095719 -4.234705 -0.197163

H -3.613413 -6.680500 -0.143115

C -6.341260 1.491175 -0.197119

H -7.411863 1.309592 -0.187601

C -5.867513 2.826862 -0.194508

C -5.461379 0.417557 -0.210988

H -5.827010 -0.604581 -0.211916

C -4.507972 3.106656 -0.205754

H -4.147959 4.130748 -0.202409

H -6.584687 3.642222 -0.183017

C 2.907569 5.919829 -0.115271

H 3.724658 6.634370 -0.093521

C 1.582129 6.389105 -0.136045

C 3.187125 4.551622 -0.121878

H 4.209503 4.188509 -0.104875

C 0.504221 5.501695 -0.164083

H -0.518666 5.863503 -0.179315

H 1.396243 7.458735 -0.129739

C 5.980803 -2.873440 -0.001216

H 6.697706 -3.688643 0.027233

C 6.454107 -1.538159 -0.010565

C 4.621051 -3.153501 -0.027802

H 4.261043 -4.177604 -0.020300

C 5.573791 -0.464812 -0.046734

H 5.939262 0.557381 -0.053674

H 7.524418 -1.355863 0.011421

N 1.497561 -3.085709 -0.097380

N -1.382920 3.037878 -0.210965

N 3.105290 1.448948 -0.127182

N -2.991456 -1.497786 -0.221031

Co 0.058150 -0.023656 -0.181783

C -1.545774 0.291090 2.628159

O -0.383928 0.611107 2.816563

H -2.358604 1.037448 2.607415

H -1.843159 -0.762698 2.492513

**6-a**

-1 3

N 0.579880 1.888237 -0.113171

C -0.267512 2.975549 -0.141270

C 1.874062 2.359197 -0.087586

C 0.502048 4.189468 -0.142582

C 1.866260 3.796925 -0.107527

N -1.828677 0.551061 -0.187492

C -2.304885 1.852471 -0.179564

C -2.916891 -0.306464 -0.168673

C -4.150003 0.479124 -0.181240

C -3.766713 1.831185 -0.192058

N -0.485697 -1.864726 -0.134826

C -1.779578 -2.338273 -0.159631

C 0.363278 -2.949045 -0.138339

C -1.769690 -3.775493 -0.187214

C -0.404235 -4.164715 -0.174866

N 1.924986 -0.526500 -0.101067

C 3.011589 0.327034 -0.078915

C 2.400366 -1.824071 -0.107226

C 3.864121 -1.807914 -0.099015

C 4.248060 -0.456660 -0.079498

C -6.450108 1.130616 -0.202528

H -7.505727 0.876988 -0.206127

C -6.066490 2.483631 -0.213056

C -5.495630 0.111700 -0.188013

H -5.791045 -0.932353 -0.180928

C -4.719215 2.850091 -0.208994

H -4.422945 3.893868 -0.218038

H -6.831907 3.253539 -0.224812

C 1.166507 6.491904 -0.163415

H 0.920475 7.549450 -0.183793

C 2.527464 6.100369 -0.129595

C 0.147896 5.547899 -0.171640

H -0.894227 5.850878 -0.199258

C 2.888427 4.759545 -0.103046

H 3.932341 4.462702 -0.078263

H 3.298218 6.865438 -0.124679

C 6.163391 -2.461839 -0.096772

H 6.928348 -3.232311 -0.102839

C 6.547663 -1.109514 -0.077168

C 4.815435 -2.827371 -0.109026

H 4.518745 -3.870986 -0.125164

C 5.593323 -0.089757 -0.069591

H 5.889513 0.954058 -0.055677

H 7.603429 -0.856510 -0.068242

C -2.424138 -6.080053 -0.249531

H -3.192764 -6.846720 -0.278429

C -1.062051 -6.468320 -0.237517

C -2.789047 -4.740094 -0.226038

H -3.833904 -4.445682 -0.237227

C -0.046029 -5.521941 -0.201402

H 0.997137 -5.822404 -0.193433

H -0.812808 -7.525126 -0.257213

N -2.915537 -1.616705 -0.155544

N 3.011801 1.638424 -0.059643

N 1.710130 -2.939234 -0.115371

N -1.614040 2.965612 -0.161891

Co 0.035081 0.013376 0.036632

C -0.405864 0.198053 1.950224

O -1.127791 -0.691092 2.615920

H -0.637413 1.219168 2.303108

H -1.741084 -0.279025 3.251498

TS12

-1 1

N -2.618917 0.646630 -0.339964

C -3.701186 -0.154649 -0.051689

C -3.010114 1.952493 -0.156450

C -4.850470 0.668504 0.297904

C -4.412186 2.003005 0.233309

N -1.445999 -1.796634 -0.723261

C -2.702029 -2.223125 -0.361292

C -0.671137 -2.915028 -0.931281

C -1.462558 -4.116703 -0.707236

C -2.749877 -3.678687 -0.348698

N 0.901060 -0.561702 -1.405755

C 1.318478 -1.870527 -1.496434

C 1.979462 0.236732 -1.694401

C 2.724907 -1.919954 -1.866990

C 3.143223 -0.583593 -2.004054

N -0.255528 1.878941 -0.957675

C -1.000564 2.995382 -0.650242

C 1.009835 2.306628 -1.283592

C 1.093118 3.756656 -1.180618

C -0.183428 4.193010 -0.784196

C -2.176352 -6.384897 -0.502065

H -1.974008 -7.450012 -0.554583

C -3.464937 -5.946480 -0.143081

C -1.158171 -5.475878 -0.790114

H -0.165105 -5.813368 -1.066730

C -3.769230 -4.587450 -0.062257

H -4.762201 -4.249789 0.214513

H -4.233711 -6.681235 0.074248

C -7.025346 1.413424 0.939164

H -8.053494 1.206068 1.218569

C -6.586554 2.749439 0.874030

C -6.163302 0.354996 0.651957

H -6.501345 -0.674399 0.703070

C -5.274046 3.062237 0.520048

H -4.935446 4.091541 0.470819

H -7.283990 3.548560 1.104453

C 1.868057 6.013228 -1.171851

H 2.658851 6.743720 -1.310161

C 0.587277 6.451462 -0.785772

C 2.139157 4.660081 -1.374540

H 3.131264 4.320687 -1.648687

C -0.455277 5.546914 -0.584547

H -1.440310 5.883276 -0.279370

H 0.412151 7.512293 -0.637006

C 4.946359 -2.660080 -2.327715

H 5.671924 -3.457409 -2.453797

C 5.364376 -1.322557 -2.466166

C 3.622083 -2.976762 -2.024889

H 3.303296 -4.006460 -1.906155

C 4.469548 -0.265362 -2.303559

H 4.794176 0.765422 -2.393838

H 6.404118 -1.113261 -2.696417

N 0.602659 -2.961238 -1.282379

N -2.270490 3.041700 -0.284683

N 2.039424 1.559412 -1.647530

N -3.750607 -1.476141 -0.055102

Co -0.827333 0.034503 -0.754684

C -0.363826 -0.046072 1.184533

O 0.547004 -1.004213 1.488597

H -0.020569 0.977324 1.372591

H -1.342196 -0.245616 1.632159

O 1.117107 -0.800289 4.115039

H 0.366307 -1.118518 4.635026

H 0.847198 -0.905682 3.165601

O 3.418549 -2.375405 4.145437

H 3.439895 -2.687302 3.212007

H 2.570170 -1.881156 4.196852

O 2.182810 2.321826 1.892598

H 2.325659 2.226543 2.864648

H 2.306279 1.388385 1.603845

O 4.596593 -0.006322 3.307968

H 4.040784 -0.013956 2.491353

H 4.318760 -0.854966 3.725071

O 2.833948 -0.285517 1.258074

H 3.145460 -0.067746 0.370049

H 1.680574 -0.617635 1.282332

O 2.633160 1.509750 4.483965

H 1.961775 0.797252 4.419537

H 3.438010 1.043167 4.147966

O 3.412511 -2.922200 1.408432

H 4.330600 -3.021651 1.119578

H 3.236304 -1.951115 1.299460

O 4.303490 2.428111 0.016303

H 3.699761 2.176666 -0.706679

H 3.682706 2.537777 0.765961

**6et**

-2 2

N 1.953257 -0.039323 -0.201437

C 2.793876 1.058366 -0.161942

C 2.743381 -1.174771 -0.176072

C 4.187932 0.612359 -0.156562

C 4.155977 -0.792528 -0.166102

N 0.050645 1.945505 -0.123476

C 1.182938 2.736153 -0.118002

C -1.043103 2.786029 -0.121769

C -0.608748 4.156842 -0.114042

C 0.811355 4.124750 -0.110673

N -1.935882 0.048886 -0.162589

C -2.726982 1.183182 -0.158027

C -2.777160 -1.049006 -0.159224

C -4.139968 0.800946 -0.177116

C -4.171705 -0.603524 -0.177020

N -0.036438 -1.938135 -0.142794

C 1.058775 -2.779272 -0.141628

C -1.167263 -2.727017 -0.131655

C -0.796086 -4.116289 -0.122939

C 0.624103 -4.149537 -0.129564

C -0.552271 6.554806 -0.108124

H -1.060661 7.514604 -0.106768

C 0.863989 6.522704 -0.104233

C -1.296096 5.381350 -0.114017

H -2.381608 5.409556 -0.118009

C 1.553716 5.316741 -0.106798

H 2.639410 5.295202 -0.105400

H 1.415329 7.458476 -0.099771

C 6.579489 0.557741 -0.132952

H 7.537549 1.068568 -0.119818

C 6.547369 -0.847886 -0.142430

C 5.398797 1.304402 -0.139487

H 5.423494 2.389414 -0.130749

C 5.333771 -1.539551 -0.158503

H 5.308375 -2.624572 -0.164299

H 7.481083 -1.402074 -0.136447

C -0.850624 -6.514141 -0.106787

H -1.402771 -7.449393 -0.097201

C 0.565558 -6.547466 -0.112745

C -1.539346 -5.307518 -0.112991

H -2.625001 -5.285007 -0.108838

C 1.310379 -5.374711 -0.125217

H 2.395876 -5.403943 -0.130553

H 1.073137 -7.507683 -0.107297

C -6.531110 0.856030 -0.205893

H -7.464914 1.409995 -0.216858

C -6.563037 -0.549621 -0.205569

C -5.317627 1.547948 -0.192189

H -5.292320 2.632981 -0.192287

C -5.382272 -1.296048 -0.191828

H -5.406723 -2.381093 -0.191525

H -7.521041 -1.060617 -0.216210

N -2.343711 2.437291 -0.131391

N 2.358081 -2.428963 -0.150880

N -2.450603 -2.319104 -0.134951

N 2.465318 2.328401 -0.124847

Co 0.016739 0.002282 -0.050201

C 0.329242 -0.044175 2.143269

O -0.792892 -0.106708 2.769259

H 0.979116 -0.937582 2.119247

H 0.904689 0.899586 2.163653

**6pt**

0 1

N -0.877171 1.745371 -0.155476

C -2.231605 1.991791 -0.128177

C -0.233691 2.962461 -0.129782

C -2.475525 3.426330 -0.103906

C -1.210812 4.040746 -0.103772

N -1.774936 -0.841879 -0.159307

C -2.990807 -0.197473 -0.137752

C -2.021115 -2.196914 -0.133632

C -3.455828 -2.439449 -0.122944

C -4.069578 -1.174164 -0.126223

N 0.811872 -1.739092 -0.167016

C 0.168754 -2.955280 -0.133346

C 2.166271 -1.982858 -0.168194

C 1.146747 -4.033035 -0.126433

C 2.411079 -3.417485 -0.149491

N 1.708063 0.852739 -0.188737

C 1.955414 2.206107 -0.163305

C 2.924391 0.207228 -0.192962

C 4.003664 1.183529 -0.194996

C 3.390455 2.448957 -0.174809

C -5.602419 -3.481568 -0.099817

H -6.223320 -4.371756 -0.088988

C -6.216900 -2.214642 -0.103051

C -4.213951 -3.611213 -0.110318

H -3.740679 -4.587148 -0.107949

C -5.459100 -1.044016 -0.116765

H -5.932408 -0.068108 -0.119250

H -7.300391 -2.151084 -0.094531

C -3.519017 5.571861 -0.059225

H -4.409575 6.192060 -0.041002

C -2.252500 6.187144 -0.059259

C -3.647774 4.183420 -0.082451

H -4.623411 3.709523 -0.082937

C -1.081428 5.430215 -0.082463

H -0.105830 5.904172 -0.083010

H -2.189597 7.270554 -0.040854

C 6.151303 2.223849 -0.202448

H 7.234774 2.160124 -0.212550

C 5.537244 3.490783 -0.182144

C 5.393134 1.053367 -0.209819

H 5.866183 0.077454 -0.225516

C 4.148745 3.620553 -0.168662

H 3.675745 4.596511 -0.153253

H 6.158390 4.380853 -0.176913

C 2.190647 -6.178802 -0.106697

H 2.128754 -7.262289 -0.089446

C 3.456430 -5.562670 -0.130103

C 1.018814 -5.422551 -0.105559

H 0.043687 -5.897190 -0.088440

C 3.583784 -4.174017 -0.153001

H 4.558931 -3.699477 -0.171752

H 4.347610 -6.182247 -0.130649

N -1.134861 -3.178170 -0.116193

N 1.069354 3.187519 -0.129299

N 3.146957 -1.095837 -0.184546

N -3.213341 1.106026 -0.120323

Co -0.031641 -0.001588 -0.068352

C -0.073188 -0.076901 1.880858

O 1.187095 -0.152750 2.483752

H -0.626392 0.818995 2.175768

H 1.607275 0.717113 2.414712

H -0.613584 -0.991723 2.122188

**TS13**

-2 4

N -2.614256 0.692176 -0.349257

C -3.718300 -0.089458 -0.048909

C -2.972532 2.015953 -0.164064

C -4.840314 0.765169 0.299472

C -4.369622 2.093522 0.228339

N -1.488542 -1.790640 -0.706918

C -2.766677 -2.185797 -0.349302

C -0.733827 -2.932089 -0.909090

C -1.560870 -4.107296 -0.693037

C -2.844387 -3.636000 -0.342772

N 0.895393 -0.609325 -1.406272

C 1.287665 -1.937180 -1.469623

C 1.998428 0.168372 -1.697340

C 2.691588 -2.013804 -1.827472

C 3.140660 -0.683053 -1.981179

N -0.213750 1.872151 -0.985608

C -0.938623 3.012891 -0.672860

C 1.066718 2.269693 -1.317035

C 1.174801 3.714845 -1.226558

C -0.094168 4.183442 -0.822462

C -2.337557 -6.363325 -0.499428

H -2.159744 -7.432975 -0.554075

C -3.618053 -5.893279 -0.149650

C -1.295816 -5.475351 -0.775790

H -0.309136 -5.838252 -1.045225

C -3.885375 -4.525206 -0.068182

H -4.872754 -4.164464 0.201656

H -4.408334 -6.607751 0.059533

C -7.000515 1.567693 0.947439

H -8.032172 1.383422 1.231196

C -6.530791 2.893181 0.875917

C -6.160740 0.489679 0.659999

H -6.523824 -0.531482 0.716645

C -5.210378 3.170217 0.515463

H -4.848796 4.192076 0.461808

H -7.207214 3.710739 1.105636

C 1.997290 5.962530 -1.243009

H 2.803363 6.673773 -1.394946

C 0.728738 6.431163 -0.849331

C 2.234654 4.600422 -1.435684

H 3.217943 4.239798 -1.716149

C -0.329472 5.546868 -0.633155

H -1.304851 5.907839 -0.323085

H 0.575446 7.496595 -0.707383

C 4.908123 -2.806971 -2.261187

H 5.616446 -3.622504 -2.370048

C 5.355293 -1.479640 -2.416337

C 3.573203 -3.087688 -1.964418

H 3.232916 -4.109649 -1.833902

C 4.478234 -0.403367 -2.275933

H 4.827014 0.618678 -2.379746

H 6.400779 -1.293633 -2.641882

N 0.550153 -3.022268 -1.247555

N -2.211598 3.099275 -0.293788

N 2.095886 1.500020 -1.675833

N -3.809117 -1.416626 -0.042948

Co -0.819448 0.032891 -0.727269

C -0.383803 -0.023298 1.191893

O 0.521060 -0.989234 1.547733

H -0.032792 0.998091 1.392178

H -1.364726 -0.198216 1.650211

O 1.110008 -0.733370 4.101847

H 0.361431 -1.032699 4.635638

H 0.826543 -0.847177 3.145182

O 3.378705 -2.337557 4.163779

H 3.402292 -2.672805 3.238743

H 2.536852 -1.828392 4.197684

O 2.229797 2.338334 1.878093

H 2.372792 2.235893 2.849446

H 2.320797 1.404581 1.585045

O 4.609071 -0.016857 3.282678

H 4.053423 -0.018872 2.468073

H 4.308839 -0.852870 3.710848

O 2.820268 -0.303478 1.241578

H 3.080404 -0.099603 0.333088

H 1.708097 -0.607548 1.299148

O 2.672704 1.529741 4.470268

H 1.991130 0.825304 4.405358

H 3.470174 1.054478 4.130261

O 3.391332 -2.948786 1.440891

H 4.307293 -3.059801 1.149436

H 3.218733 -1.981885 1.305227

O 4.323451 2.323183 -0.056250

H 3.670511 2.065062 -0.741449

H 3.744419 2.480015 0.716156

**7**

-1 2

N -0.404013 1.917170 -0.154177

C -1.654398 2.502211 -0.135592

C 0.531016 2.932472 -0.130233

C -1.524155 3.932533 -0.119044

C -0.132173 4.206486 -0.114466

N -1.935506 -0.362714 -0.167876

C -2.952229 0.577322 -0.139406

C -2.521435 -1.617816 -0.124220

C -3.976597 -1.478763 -0.104822

C -4.246935 -0.101254 -0.117177

N 0.342109 -1.893958 -0.173352

C -0.591912 -2.908684 -0.136185

C 1.593109 -2.476127 -0.185295

C 0.072269 -4.182333 -0.138776

C 1.463871 -3.906766 -0.171199

N 1.873637 0.391031 -0.198410

C 2.461167 1.644589 -0.157842

C 2.891065 -0.550442 -0.200634

C 4.186151 0.127462 -0.189558

C 3.916658 1.505007 -0.159884

C -6.321157 -1.939754 -0.067949

H -7.147621 -2.643507 -0.048475

C -6.591960 -0.559581 -0.080499

C -5.008963 -2.416441 -0.080677

H -4.799129 -3.480953 -0.071642

C -5.557051 0.377218 -0.105731

H -5.764994 1.442090 -0.115641

H -7.623022 -0.219870 -0.070636

C -1.986004 6.283160 -0.090038

H -2.687992 7.111650 -0.079839

C -0.595664 6.556816 -0.085836

C -2.461058 4.978706 -0.107396

H -3.526638 4.771568 -0.111411

C 0.338325 5.529666 -0.099022

H 1.402885 5.741963 -0.096509

H -0.259886 7.589425 -0.072425

C 6.531712 0.584849 -0.171697

H 7.562686 0.244742 -0.174864

C 6.261631 1.964940 -0.141825

C 5.496277 -0.351295 -0.196738

H 5.703703 -1.416080 -0.219067

C 4.949571 2.442087 -0.136474

H 4.740243 3.506489 -0.113251

H 7.088542 2.668166 -0.122364

C 0.538493 -6.532271 -0.126264

H 0.203971 -7.565213 -0.108181

C 1.928054 -6.257223 -0.159132

C -0.396520 -5.505884 -0.116971

H -1.460598 -5.719239 -0.092502

C 2.401485 -4.952198 -0.182898

H 3.466578 -4.744064 -0.208089

H 2.630918 -7.085012 -0.165605

N -1.930413 -2.786469 -0.105414

N 1.870220 2.812947 -0.122211

N 2.785406 -1.855434 -0.199533

N -2.847249 1.882727 -0.125774

Co -0.031972 0.005856 -0.059025

C -0.094653 -0.056016 1.875656

O 1.107003 -0.450734 2.492732

H -0.401549 0.949841 2.180975

H 1.762495 0.240972 2.322011

H -0.850656 -0.798357 2.133478

**7et**

-2 1

N -0.223510 1.952310 -0.155949

C -1.418809 2.652340 -0.148658

C 0.805287 2.879007 -0.136359

C -1.158598 4.046075 -0.144313

C 0.268507 4.191360 -0.136595

N -1.966130 -0.184918 -0.204266

C -2.895484 0.852243 -0.147019

C -2.668412 -1.387438 -0.135076

C -4.108583 -1.106732 -0.095200

C -4.250056 0.288593 -0.105276

N 0.167534 -1.927236 -0.169971

C -0.860532 -2.853819 -0.151076

C 1.363690 -2.624335 -0.180124

C -0.322992 -4.165741 -0.165351

C 1.104078 -4.018379 -0.184368

N 1.911436 0.214891 -0.178359

C 2.615943 1.417018 -0.142631

C 2.842067 -0.824156 -0.174661

C 4.197125 -0.260569 -0.160845

C 4.056641 1.135035 -0.139037

C -6.488576 -1.348755 -0.022041

H -7.375374 -1.974623 0.010706

C -6.630276 0.049067 -0.032312

C -5.223619 -1.942680 -0.053808

H -5.113100 -3.022405 -0.045882

C -5.510178 0.884276 -0.074239

H -5.618574 1.964207 -0.081809

H -7.624755 0.484486 -0.007248

C -1.398369 6.440296 -0.137667

H -2.019936 7.331528 -0.136953

C 0.020851 6.584805 -0.131855

C -1.988662 5.189492 -0.144710

H -3.070398 5.086037 -0.150173

C 0.851092 5.478542 -0.132118

H 1.931450 5.595145 -0.128058

H 0.449891 7.583082 -0.127300

C 6.578650 -0.022016 -0.143543

H 7.573253 -0.457870 -0.144222

C 6.437862 1.375855 -0.121778

C 5.457603 -0.856532 -0.164326

H 5.565271 -1.936428 -0.181322

C 5.172864 1.970310 -0.120429

H 5.063056 3.050021 -0.104185

H 7.325481 2.001216 -0.106082

C -0.072715 -6.558816 -0.176282

H -0.500586 -7.557584 -0.172426

C 1.346092 -6.412484 -0.196206

C -0.904097 -5.453406 -0.162228

H -1.984248 -5.571212 -0.147870

C 1.935050 -5.161004 -0.201367

H 3.016569 -5.056393 -0.216579

H 1.968668 -7.302953 -0.207258

N -2.201869 -2.599770 -0.112631

N 2.148583 2.628848 -0.118612

N 2.627223 -2.104990 -0.178302

N -2.681924 2.133447 -0.127512

Co -0.034262 0.006270 -0.042051

C -0.139031 -0.045831 1.878099

O 1.045601 -0.478696 2.521985

H -0.414956 0.970777 2.184563

H 1.758024 0.089819 2.195285

H -0.925351 -0.754349 2.145930

**TS14**

-1 2

N 0.671762 -1.816434 -1.088630

C 1.734976 -2.665246 -0.860102

C -0.443426 -2.589487 -1.277952

C 1.284151 -4.048462 -0.919334

C -0.099011 -3.999416 -1.175216

N 2.626223 0.058771 -0.621202

C 3.389310 -1.076599 -0.486720

C 3.431784 1.139500 -0.349723

C 4.788440 0.688028 -0.062790

C 4.761974 -0.716009 -0.151707

N 0.758487 2.055168 -0.908825

C 1.848135 2.826026 -0.564509

C -0.305954 2.899083 -1.097982

C 1.465381 4.230340 -0.519281

C 0.099596 4.275893 -0.857529

N -1.189915 0.183759 -1.404652

C -2.007129 -0.903344 -1.638695

C -1.934521 1.326874 -1.627630

C -3.288028 0.962147 -2.017440

C -3.339850 -0.446519 -2.005349

C 7.111656 0.609690 0.482998

H 8.042851 1.108612 0.732478

C 7.085231 -0.793931 0.394189

C 5.962596 1.369399 0.256575

H 5.982426 2.451724 0.327405

C 5.908987 -1.475634 0.077186

H 5.888334 -2.558262 0.011486

H 7.996492 -1.354658 0.577150

C 1.182329 -6.432723 -0.839496

H 1.663336 -7.397157 -0.710180

C -0.201787 -6.382509 -1.091189

C 1.942978 -5.266589 -0.749617

H 3.008475 -5.305934 -0.549723

C -0.860955 -5.165076 -1.262622

H -1.928750 -5.124936 -1.449016

H -0.764348 -7.308997 -1.149195

C -5.597674 1.041823 -2.619016

H -6.494658 1.603510 -2.859317

C -5.651929 -0.365152 -2.600884

C -4.417146 1.723405 -2.324909

H -4.378038 2.807266 -2.328239

C -4.524477 -1.127982 -2.294844

H -4.566186 -2.211641 -2.275766

H -6.589439 -0.863093 -2.826487

C 1.482736 6.605287 -0.271046

H 2.004117 7.529985 -0.044454

C 0.116931 6.650403 -0.609335

C 2.174802 5.394870 -0.222865

H 3.227372 5.360024 0.037381

C -0.592451 5.486833 -0.907728

H -1.645025 5.521711 -1.167932

H -0.391181 7.609218 -0.637076

N 3.081612 2.415245 -0.314339

N -1.682301 -2.180793 -1.541507

N -1.544304 2.577468 -1.463465

N 2.988658 -2.333823 -0.597222

Co 0.717534 0.117310 -0.927963

C 0.306909 0.044059 1.473066

O -1.122666 0.187215 1.439824

H 0.705853 -0.933195 1.253830

H -1.407113 0.112449 0.509881

H 0.880401 0.957243 1.536167

O -3.256229 -1.035041 2.547328

H -2.383211 -0.666642 2.265892

H -3.902988 -0.516453 1.998749

O -4.731372 0.562500 0.964881

H -4.049362 1.273384 1.022159

H -4.567738 0.141312 0.108839

O -2.714227 2.402937 1.415952

H -2.521884 2.873189 0.589524

H -1.991905 1.740044 1.505850

O 0.146979 -1.009420 4.003985

H 0.343921 -0.556263 3.086565

H 1.012396 -1.137510 4.419929

O -0.175761 -3.281231 2.164897

H -0.183823 -2.568681 2.829662

H -1.075291 -3.274844 1.772875

O -0.701071 1.706180 4.460233

H -1.674362 1.657001 4.361331

H -0.421230 0.772966 4.428235

O -2.763626 -3.304307 1.038644

H -3.035139 -2.509752 1.551897

**TS15**

-2 3

N 0.700944 -1.871004 -1.049995

C 1.716975 -2.769391 -0.795680

C -0.449900 -2.589712 -1.263884

C 1.206314 -4.112909 -0.863906

C -0.178493 -3.997953 -1.156808

N 2.721349 -0.080269 -0.478614

C 3.436047 -1.259524 -0.354518

C 3.566789 0.965787 -0.179938

C 4.901944 0.444448 0.127128

C 4.820943 -0.953622 0.010672

N 0.958567 2.013135 -0.834467

C 2.071180 2.729575 -0.464419

C -0.057356 2.913376 -1.091928

C 1.769420 4.138492 -0.470969

C 0.412722 4.254042 -0.872905

N -1.057354 0.231290 -1.436426

C -1.934134 -0.820495 -1.630269

C -1.759191 1.415347 -1.643451

C -3.140592 1.102321 -1.995438

C -3.259161 -0.299648 -1.954410

C 7.214427 0.263677 0.706478

H 8.160143 0.721740 0.979567

C 7.133488 -1.134576 0.590946

C 6.097261 1.070748 0.475390

H 6.158503 2.150418 0.565590

C 5.933902 -1.760582 0.242055

H 5.871460 -2.840399 0.154291

H 8.018056 -1.736080 0.776836

C 0.983648 -6.498466 -0.786005

H 1.413402 -7.485812 -0.644889

C -0.397944 -6.381916 -1.071560

C 1.793601 -5.374074 -0.679198

H 2.851380 -5.468022 -0.453368

C -0.989338 -5.138166 -1.258917

H -2.050526 -5.049016 -1.469942

H -1.002260 -7.281467 -1.141454

C -5.469904 1.276195 -2.518047

H -6.346828 1.875759 -2.740761

C -5.590001 -0.125164 -2.455204

C -4.244673 1.907164 -2.285096

H -4.157200 2.988035 -2.315693

C -4.484384 -0.929373 -2.174353

H -4.575665 -2.008524 -2.110938

H -6.558872 -0.583585 -2.626009

C 1.915849 6.523649 -0.285718

H 2.480422 7.423944 -0.062066

C 0.562175 6.638325 -0.683733

C 2.528854 5.280908 -0.177385

H 3.567079 5.195068 0.128151

C -0.197778 5.513417 -0.980284

H -1.235426 5.604789 -1.286190

H 0.113956 7.624771 -0.757754

N 3.291641 2.250416 -0.151222

N -1.680017 -2.105663 -1.534243

N -1.318163 2.640342 -1.482610

N 2.998684 -2.487571 -0.493852

Co 0.815219 0.066396 -0.845382

C 0.185603 -0.084491 1.492697

O -1.203025 0.313538 1.391416

H 0.415280 -1.118869 1.298202

H -1.393892 0.362887 0.437644

H 0.889423 0.692835 1.749852

O -3.623373 -0.715904 1.943375

H -2.677410 -0.454063 1.821483

H -4.134998 0.009023 1.497413

O -4.800631 1.465567 0.889158

H -3.970327 1.985549 0.994221

H -4.911017 1.386133 -0.070128

O -2.385324 2.774304 1.397716

H -2.182181 3.044699 0.485198

H -1.838271 1.965112 1.527454

O -0.400445 -1.162231 3.958546

H -0.037903 -0.752593 3.065794

H 0.364961 -1.571568 4.388322

O -0.994098 -3.353916 2.074812

H -0.980809 -2.626280 2.721546

H -1.835499 -3.249548 1.581325

O -0.823827 1.566748 4.557204

H -1.781318 1.616595 4.358985

H -0.634894 0.611982 4.473810

O -3.366790 -3.085487 0.549237

H -3.562827 -2.223129 0.981177

H -2.885703 -2.832416 -0.267742

O -3.548867 1.664720 3.841448

H -3.363826 2.258866 3.095479

H -3.585319 0.787105 3.422009

O -3.093400 -1.633016 4.536528

H -2.136481 -1.478175 4.410042

H -3.456590 -1.392246 3.664268

## Reference

[1] a) P. Hutchison, L. E. Smith, C. L. Rooney, H. Wang, S. Hammes-Schiffer, *J. Am. Chem. Soc.* **2024**, *146*, 20230-20240; b) L. L. Shi, M. Li, B. You, R. Z. Liao, *Inorg. Chem.* **2022**, *61*, 16549-16564.

[2] Y. Wu, Z. Jiang, X. Lu, Y. Liang, H. Wang, *Nature* **2019**, *575*, 639-642.

[3] M. Frisch, *Revision B* **2016**, *1*.

[4] a) S. Grimme, J. Antony, S. Ehrlich, H. Krieg, *J. Chem. Phys.* **2010**, *132*; b) A. Becke, *Chem. Phys*, *98*, 5648.

[5] A. V. Marenich, C. J. Cramer, D. G. Truhlar, *J. Phys. Chem. B* **2009**, *113*, 6378-6396.

[6] D. Andrae, U. Haeussermann, M. Dolg, H. Stoll, H. Preuss, *Theor. Chim. Acta* **1990**, *77*, 123-141.

[7] a) C. J. Cramer, J. A. Bumpus, A. Lewis, C. Stotts, *J. Chem. Educ.* **2007**, *84*, 329; b) D. G. Truhlar, C. J. Cramer, A. Lewis, J. A. Bumpus, *J. Chem. Educ.* **2004**, *81*, 596.

[8] A. A. Isse, A. Gennaro, *J. Phys. Chem. B* **2010**, *114*, 7894-7899.

[9] a) H. J. C. Berendsen, D. van der Spoel, R. van Drunen, *Comput. Phys. Commun.* **1995**, *91*, 43-56; b) M. J. Abraham, T. Murtola, R. Schulz, S. Páll, J. C. Smith, B. Hess, E. Lindahl, *SoftwareX* **2015**, *1*, 19-25.

[10] T. Lu, F. Chen, *J. Comput. Chem.* **2012**, *33*, 580-592.

[11] W. L. Jorgensen, D. S. Maxwell, J. Tirado-Rives, *J. Am. Chem. Soc.* **1996**, *118*, 11225-11236.

[12] S. Tian Lu, Version [Version 1.0], <http://sobereva.com/soft/Sobtop> (accessed on Dec. 26, 2023).

[13] a) W. L. Jorgensen, *ChemInform* **2002**, *12*; b) J.-P. Ryckaert, G. Ciccotti, H. J. C. Berendsen, *J. Comput. Phys.* **1977**, *23*, 327-341; cA. Alexiadis, S. Kassinos, *Chem. Rev.* **2008**, *108*, 5014-5034.

[14] J. A. de Gracia Triviño, M. S. G. Ahlquist, *ACS Catal.* **2023**, *13*, 1270-1279.

[15] E. Boutin, M. Wang, J. C. Lin, M. Mesnage, D. Mendoza, B. Lassalle-Kaiser, C. Hahn, T. F. Jaramillo, M. Robert, *Angew. Chem. Int. Ed.* **2019**, *58*, 16172-16176.
